# Stage‐Adaptive Bilayer Hydrogel Coordinates Photodynamic Antibacterial Therapy with Engineered Extracellular Vesicle–Mediated Immunomodulation in Infected Diabetic Wounds

**DOI:** 10.1002/advs.78491

**Published:** 2026-11-10

**Authors:** Gaofei Zhang, Boheng Zhu, Shihao Deng, Pan Du, Jun Pu, Xin Wang, Shuqian Dou, Yifei Wang, Wenjun Liu

**Affiliations:** ^1^ The Second Affiliated Hospital of Kunming Medical University Kunming Yunnan China; ^2^ Kunming Medical University Kunming Yunnan China

**Keywords:** bilayer hydrogel, engineered small extracellular vesicles, infected diabetic wound, IRAK1/NF‐κB, macrophage immunoregulation, miR‐146a, photodynamic therapy

## Abstract

During the early phase of treatment, infected diabetic wounds require rapid bacterial control, whereas subsequent healing benefits from relief of persistent oxidative stress and inflammation, resulting in distinct and partly opposing redox demands over time. We therefore developed a stage‐adaptive bilayer hydrogel that separates photodynamic antibacterial therapy from engineered small extracellular vesicle (sEV) delivery. The wound‐contacting layer contains chlorin e6 (Ce6), while the ROS‐responsive upper layer scavenges ROS and retains miR‐146a‐enriched sEVs carrying a human CD64‐recognition module. The bilayer architecture limited premature release of sEV‐associated particles during photodynamic treatment but allowed substantial release thereafter, with key vesicle‐associated features preserved after irradiation. In human macrophages, engineered sEVs increased intracellular miR‐146a, reduced IRAK1 abundance and NF‐κB p65 phosphorylation, decreased CD86, and increased CD206. These effects were partly weakened by CD64 blockade. In infected diabetic wounds, Bil‐Gel@miR‐sEVs reduced bacterial burden and oxidative stress, suppressed IRAK1/NF‐κB‐associated inflammatory signaling, shifted macrophage‐associated markers toward a repair‐associated pattern, and improved epithelial, vascular, and matrix reconstruction. This bilayer design therefore links early infection control with later immunoregulation and tissue repair within one biomaterial.

## Graphical abstract

Early photodynamic infection control and subsequent immunoregulatory repair are spatially separated within a stage‐adaptive bilayer hydrogel. Chlorin e6 is incorporated into the wound‐contacting layer to provide light‐triggered bactericidal therapy, whereas the upper layer regulates excessive reactive oxygen species before releasing microRNA‐146a‐enriched engineered small extracellular vesicles. Through this functional compartmentalization, antibacterial activity and oxidative‐stress regulation are temporally coordinated with macrophage immunomodulation, angiogenesis, and tissue reconstruction in infected diabetic wounds.

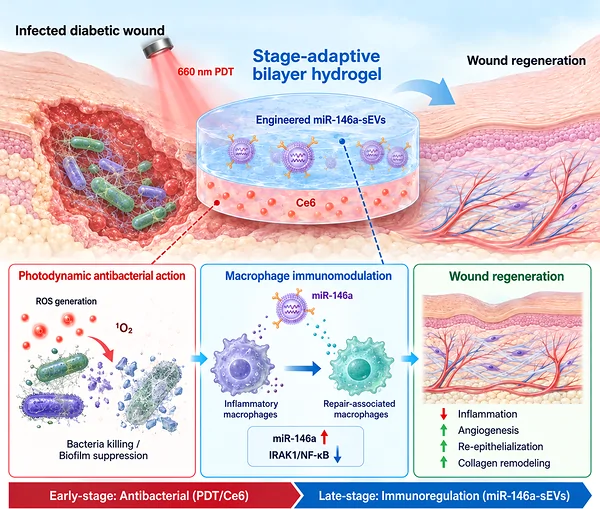

## 1 Introduction

Infected diabetic wounds are challenging to treat because bacterial colonization and biofilm formation develop within a hyperglycemic microenvironment already impaired by vascular dysfunction, excessive oxidative stress, compromised innate immunity, and defective tissue regeneration [1, 2, 3]. Whereas acute wounds progress through coordinated phases of hemostasis, inflammation, proliferation, and remodeling, diabetic wounds frequently remain locked in prolonged inflammation [4, 5]. Infection further intensifies biofilm formation and immune dysregulation [6, 7, 8], and excessive reactive oxygen species (ROS) amplify inflammatory signaling and cellular dysfunction [9, 10, 11]. To protect cells involved in tissue repair, recent hydrogel‐based approaches have incorporated ROS‐responsive or antioxidant components [12, 13, 14, 15]. However, because antibacterial treatment and tissue repair do not necessarily require the same local conditions, effective therapy may depend on temporal control of the wound microenvironment.

Macrophages play a central role in the transition from inflammation to repair [16, 17]. Persistent inflammatory signaling and dysregulated macrophage responses in diabetic wounds interfere with tissue reconstruction [18, 19, 20, 21]. As an endogenous negative‐feedback regulator of innate immune signaling, microRNA‐146a (miR‐146a) suppresses interleukin‐1 receptor‐associated kinase 1 (IRAK1) and related signaling adaptors [22, 23]. In diabetic wound and other inflammatory models, extracellular‐vesicle‐mediated delivery of miR‐146a has been reported to attenuate IRAK1‐associated inflammatory signaling [24, 25, 26]. Accordingly, local miR‐146a delivery offers an attractive means of modulating the persistent inflammation characteristic of infected diabetic wounds.

Small extracellular vesicles (sEVs) derived from mesenchymal stromal cells can protect regulatory RNA cargo against extracellular degradation while facilitating intracellular delivery [27, 28]. Because of their endogenous pro‐regenerative cargo, relative accessibility, and close involvement in hemostatic and reparative processes, platelet‐ and other blood‐derived extracellular vesicles (EVs) have also attracted considerable interest in wound repair [29, 30]. For the engineering strategy used here, however, a renewable and readily modifiable producer‐cell source was particularly important. Human umbilical cord‐derived mesenchymal stromal cells (hUC‐MSCs) can be obtained non‐invasively from umbilical cords routinely discarded after delivery and expanded in vitro, providing a sustainable source of producer cells. Their high amenability to genetic engineering also allows defined RNA cargo and receptor‐recognition modules to be introduced during vesicle biogenesis. These properties made hUC‐MSC‐derived sEVs particularly suitable for a design requiring simultaneous miR‐146a enrichment and incorporation of a human CD64‐recognition module. Hydrogel‐based delivery of native, preconditioned, and engineered EVs has been explored in diabetic and chronic wound models to improve local retention and support tissue repair [31, 32, 33, 34, 35, 36].

Producer‐cell engineering, however, increases manufacturing complexity and imposes greater demands on production yield and batch‐to‐batch consistency [37, 38]. We therefore enriched miR‐146a in hUC‐MSC‐derived sEVs using an MS2/pac strategy and incorporated a single‐chain variable fragment specific for human CD64 as the macrophage‐recognition module. Gaussia luciferase was included only as a reporter.

The changing redox state of the wound must also be accommodated by the delivery matrix. Photodynamic antibacterial therapy requires transient ROS generation at the wound interface [39, 40, 41, 42, 43], whereas persistent oxidative stress during the later repair phase is detrimental to immune regulation and tissue reconstruction [9, 10, 11]. Although antibacterial, photoactive [44, 45, 46, 47], and immunoregulatory hydrogels have each shown value in wound treatment [48], combining these functions within a single compartment risks exposing biological cargo to the oxidative conditions required for bacterial killing. We therefore spatially separated these functions within a bilayer hydrogel. Chlorin e6 was incorporated into a dynamic phenylboronic acid‐functionalized hyaluronic acid (HA‐PBA)/poly(vinyl alcohol) (PVA) wound‐contacting layer, whereas miR‐146a‐enriched engineered sEVs were placed within a distinct ROS‐responsive poly(ethylene glycol) (PEG) upper layer. By forming the two compartments independently before assembly, we preserved separation between early photodynamic treatment and subsequent vesicle delivery.

Following 660 nm irradiation, early bacterial control is provided by the lower layer, which subsequently undergoes progressive dissociation and degradation. The upper layer is designed to attenuate persistent oxidative stress while supporting local delivery of engineered sEVs. Following cellular uptake, enriched miR‐146a is expected to reduce IRAK1 abundance and p65 phosphorylation, thereby attenuating nuclear factor kappa B (NF‐κB)‐associated inflammatory signaling and promoting a repair‐associated macrophage phenotype. These changes are expected to support angiogenesis, re‐epithelialization, collagen organization, and extracellular‐matrix remodeling (Scheme 1).

**SCHEME 1:**
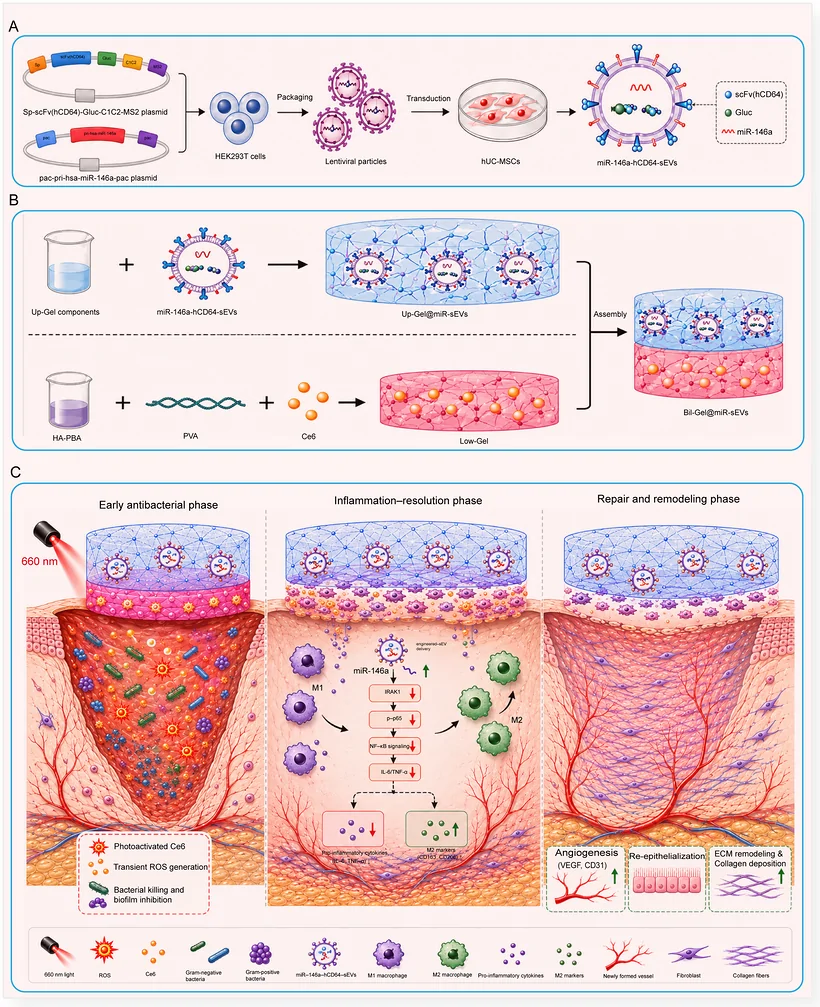
Construction of engineered sEVs, fabrication of the functionally compartmentalized bilayer hydrogel, and proposed mechanism for infected diabetic wound repair. (A) Schematic illustration of the generation of miR‐146a‐hCD64‐sEVs. The Sp‐scFv(hCD64)‐Gluc‐C1C2‐MS2 and pac‐pri‐hsa‐miR‐146a‐pac plasmids were used to produce lentiviral particles in HEK293T cells. Following transduction of hUC‐MSCs, the modified cells secreted miR‐146a‐enriched engineered sEVs carrying a human CD64‐recognition module and a Gluc reporter. (B) Fabrication of the bilayer hydrogel dressing. Engineered sEVs were incorporated into a ROS‐responsive, thioketal‐containing PEG network to form Up‐Gel@miR‐sEVs. HA‐PBA, PVA, and Ce6 were combined separately to form the wound‐contacting Low‐Gel. The two independently prepared layers were then assembled into Bil‐Gel@miR‐sEVs, preserving spatial separation between the photodynamic antibacterial compartment and the sEV‐delivery compartment. (C) Proposed stage‐adaptive functions of Bil‐Gel@miR‐sEVs in infected diabetic wounds. During the early antibacterial phase, Ce6 in the lower Low‐Gel compartment is photoactivated under 660 nm irradiation to generate a transient local ROS burst, leading to bacterial damage and biofilm suppression. Following the photodynamic phase, oxidation‐triggered cleavage of thioketal (TK) linkages in the ROS‐responsive upper Up‐Gel compartment consumes and buffers excessive ROS, while concomitant network relaxation and degradation provide a responsive material basis for the subsequent local release and delivery of engineered sEVs. Enriched miR‐146a reduces IRAK1 abundance and p65 phosphorylation, thereby attenuating NF‐κB signaling and pro‐inflammatory cytokine expression while promoting a repair‐associated macrophage phenotype. These temporally coordinated effects support angiogenesis, re‐epithelialization, collagen organization, and extracellular‐matrix remodeling, ultimately accelerating infected diabetic wound repair. Schematic panel A was assembled using BioRender.com; ChatGPT (OpenAI; GPT‐5.6 Sol) assisted concept development and visual refinement.

We examined engineered‐sEV production and cargo enrichment, bilayer structure and mechanics, temporal release of vesicle‐associated particles, preservation of vesicle‐associated characteristics during photodynamic treatment, antibacterial and antibiofilm activity, macrophage signaling, and repair‐associated responses both in vitro and in an infected diabetic wound model. Specifically, we asked whether spatial compartmentalization could temporally separate the transient oxidative conditions needed for early bacterial control from the subsequent microenvironment required for immunoregulation and tissue repair.

## 2 Results and Discussion

### 2.1 Identification of miR‐146a and Construction of Engineered sEVs with a Human CD64‐Recognition Module

To identify inflammation‐responsive microRNAs (miRNAs) for subsequent vesicle engineering, we performed small‐RNA sequencing on sEVs derived from untreated andlipopolysaccharide (LPS)‐preconditioned human umbilical cord mesenchymal stromal cells (hUC‐MSCs). Figure S1 summarizes the sequencing workflow, global miRNA profiles, representative differentially expressed miRNAs, and quantitative reverse transcription polymerase chain reaction (qRT‐PCR) validation. Among the candidates examined, miR‐146a‐5p showed enrichment in sEVs after LPS preconditioning by both sequencing and qRT‐PCR and was therefore selected as the regulatory cargo.

For simultaneous miR‐146a enrichment and incorporation of a human CD64‐recognition module, we established an MS2/pac‐based engineering system comprising the Sp‐scFv(hCD64)‐Gluc‐C1C2‐MS2 and PAC‐pri‐hsa‐miR‐146a‐PAC constructs (Figure 1A). The pac sequences flanking pri‐hsa‐miR‐146a are recognized by the MS2 RNA‐binding domain, whereas the C1C2 domain promotes association of the fusion protein with the vesicular membrane. We incorporated a single‐chain variable fragment directed against human CD64 as the receptor‐recognition moiety and Gaussia luciferase (Gluc) was included as a bioluminescent reporter. Sequences S1 and S2 in the s provide the verified coding sequence of Sp‐scFv(hCD64)‐Gluc‐C1C2‐MS2 and the Sanger‐confirmed region of the PAC‐pri‐hsa‐miR‐146a‐PAC construct, respectively.

**FIGURE 1:**
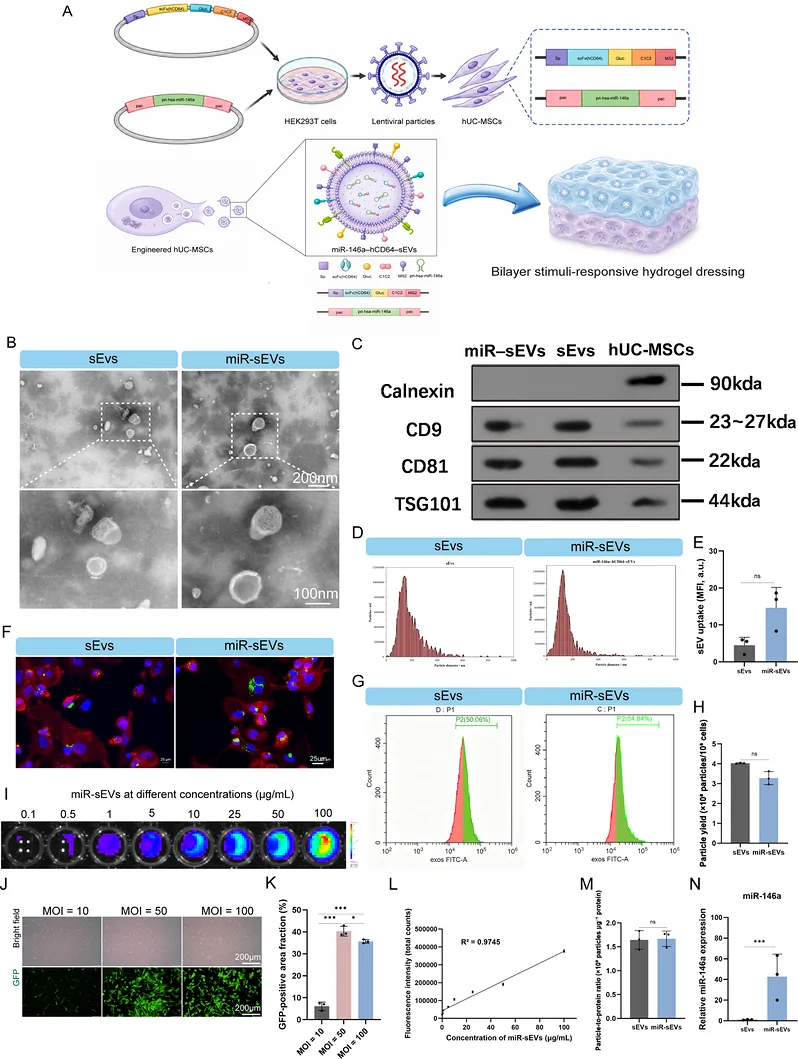
Construction and characterization of miR‐146a‐enriched engineered sEVs bearing a human CD64‐recognition module. (A) Schematic of the MS2/pac‐based engineering strategy used to generate miR‐146a‐enriched engineered sEVs bearing a human CD64‐recognition module (miR‐sEVs), followed by their incorporation into the bilayer hydrogel system. (B) Representative TEM images of sEVs and miR‐sEVs at two magnifications (scale bars: 200 and 100 nm). (C) Western blot analysis of the EV‐associated proteins CD9, CD81, and TSG101 and the non‐EV‐associated cellular protein Calnexin in sEVs, miR‐sEVs, and hUC‐MSC lysates. (D) Representative NTA particle‐size distributions of sEVs and miR‐sEVs. (E) Mean fluorescence intensity following cellular uptake of fluorescently labeled sEVs and miR‐sEVs. (F) Representative fluorescence images of cellular uptake (scale bar: 25 µm). (G) Representative flow‐cytometry profiles of cellular uptake. (H) Cell‐normalized particle yields of sEVs and miR‐sEVs. (I) Representative Gaussia luciferase (Gluc)‐associated bioluminescence images at the indicated miR‐sEV protein concentrations. J) Representative bright‐field and GFP fluorescence images of hUC‐MSCs transduced at the indicated multiplicities of infection (scale bar: 200 µm). (K) Quantification of GFP‐positive area. (L) Linear relationship between miR‐sEV protein concentration and Gluc‐associated bioluminescence (R^2^ = 0.9745). (M) Particle‐to‐protein ratios of sEVs and miR‐sEVs, expressed as particles per µg of total vesicle protein. (N) Relative miR‐146a enrichment in miR‐sEVs compared with sEVs; statistical inference was performed using ΔCt values. Quantitative data are presented as mean ± SD. For E, n = 3 independent experiments per group; groups were compared using a two‐sided unpaired Student's t‐test. For N, n = 3 independently prepared vesicle batches per group; statistical testing was performed on ΔCt values using a two‐sided unpaired Student's t‐test. For H and M, n = 3 independent production batches per group; H was analyzed using a two‐sided Welch's t‐test and M using a two‐sided unpaired Student's t‐test. For K, n = 3 independent experiments per condition; data were analyzed by one‐way ANOVA followed by Tukey's post hoc test. ^*^ *p* < 0.05, ^**^ *p* < 0.01, ^***^ *p* < 0.001; ns, not significant. Schematic panel A was created using BioRender.com.

The two constructs were introduced into hUC‐MSCs by co‐transduction with recombinant lentiviral particles. Across all tested multiplicities of infection, green fluorescent protein (GFP) fluorescence was detectable, with the largest GFP‐positive area observed at a multiplicity of infection (MOI) of 50 (Figure 1J,K); this condition was therefore selected for subsequent production of engineered sEVs, hereafter termed miR‐sEVs. As miR‐sEV concentration increased, Gluc‐associated bioluminescence also increased (Figure 1I), while the corresponding calibration curve remained linear across the tested range (R^2^ = 0.9745; Figure 1L). This concentration‐dependent reporter response supported successful generation of the engineered vesicle preparation and demonstrated that Gluc signal intensity tracked miR‐sEV abundance over the examined range.

We next assessed miR‐146a enrichment across independent preparations by qRT‐PCR. Across three independently prepared batches per group, miR‐sEVs consistently showed greater miR‐146a abundance than sEVs (Figure 1N), indicating reproducible enrichment of the intended RNA cargo. Because miR‐146a enrichment occurs during vesicle biogenesis in the MS2/pac system rather than through post‐isolation loading, these measurements reflect relative cargo enrichment among independently prepared vesicle batches rather than conventional encapsulation efficiency.

The principal vesicular characteristics of the preparations were not materially altered by engineering. In both sEV and miR‐sEV samples, transmission electron microscopy (TEM) showed rounded or cup‐shaped nanoscale vesicles (Figure 1B). Both preparations contained CD9, CD81, and TSG101, whereas Calnexin was absent from the vesicle fractions and detected only in hUC‐MSC lysates (Figure 1C). Similarly, nanoparticle tracking analysis (NTA) revealed comparable particle‐size distributions between sEVs and miR‐sEVs (Figure 1D).

Using three independently prepared batches per group, we next assessed particle production. Cell‐normalized yields were 4.03 ± 0.03 × 10^8^ particles per 10^6^ cells for sEVs and 3.28 ± 0.33 × 10^8^ particles per 10^6^ cells for miR‐sEVs, with no statistically significant difference between groups (two‐sided Welch's t‐test, *p* = 0.059; Figure 1H).

For a comparative purity‐related measure, particle‐to‐protein ratios were 1.64 ± 0.19 × 10^8^ particles µg−^1^ protein for sEVs and 1.67 ± 0.16 × 10^8^ particles µg−^1^ protein for miR‐sEVs, with no significant difference between groups (two‐sided unpaired Student's t‐test, *p* = 0.872; Figure 1M). Considered together with the conserved EV‐associated marker profile and absence of detectable Calnexin in the vesicle preparations, these findings indicate that the engineering procedure did not measurably alter the particle‐to‐protein characteristics of the isolated vesicles.

After engineering, cellular uptake was likewise retained. Following exposure to either sEVs or miR‐sEVs, fluorescence microscopy and flow cytometry detected intracellular vesicle‐associated signals (Figure 1F,G). Mean fluorescence intensity was higher in the miR‐sEV group than in the sEV group, consistent with a trend toward greater internalization, although this difference did not reach statistical significance (Figure 1E).

Collectively, the MS2/pac‐based strategy produced miR‐146a‐enriched sEVs bearing a human CD64‐recognition module while preserving the major physicochemical characteristics, particle‐to‐protein profile, and cellular uptake capacity of the vesicle preparations.

### 2.2 Fabrication and Physicochemical Characterization of the Functionally Compartmentalized Bilayer Hydrogel

To spatially separate photodynamic antibacterial activity from engineered‐sEV delivery, we designed a functionally compartmentalized bilayer hydrogel. The wound‐contacting Low‐Gel contained Ce6 to provide localized photodynamic treatment, whereas the upper Up‐Gel functioned as a physically distinct reservoir for engineered sEVs. By confining the early ROS‐dependent antibacterial function to the wound interface and reserving the upper compartment for subsequent vesicle delivery, this arrangement accommodated the different microenvironmental requirements of the two therapeutic phases.

Low‐Gel was generated through reversible boronate‐ester interactions between HA‐PBA and PVA, with Ce6 incorporated as the photosensitizer (Figure 2A,B). After HA‐PBA and PVA were mixed, rapid gelation was evident in the vial‐inversion assay (Figure 2B). PBA functionalization of HA was confirmed by FTIR and 1H NMR analyses (Figure 2E,G), supporting formation of the dynamic boronate‐ester network.

**FIGURE 2:**
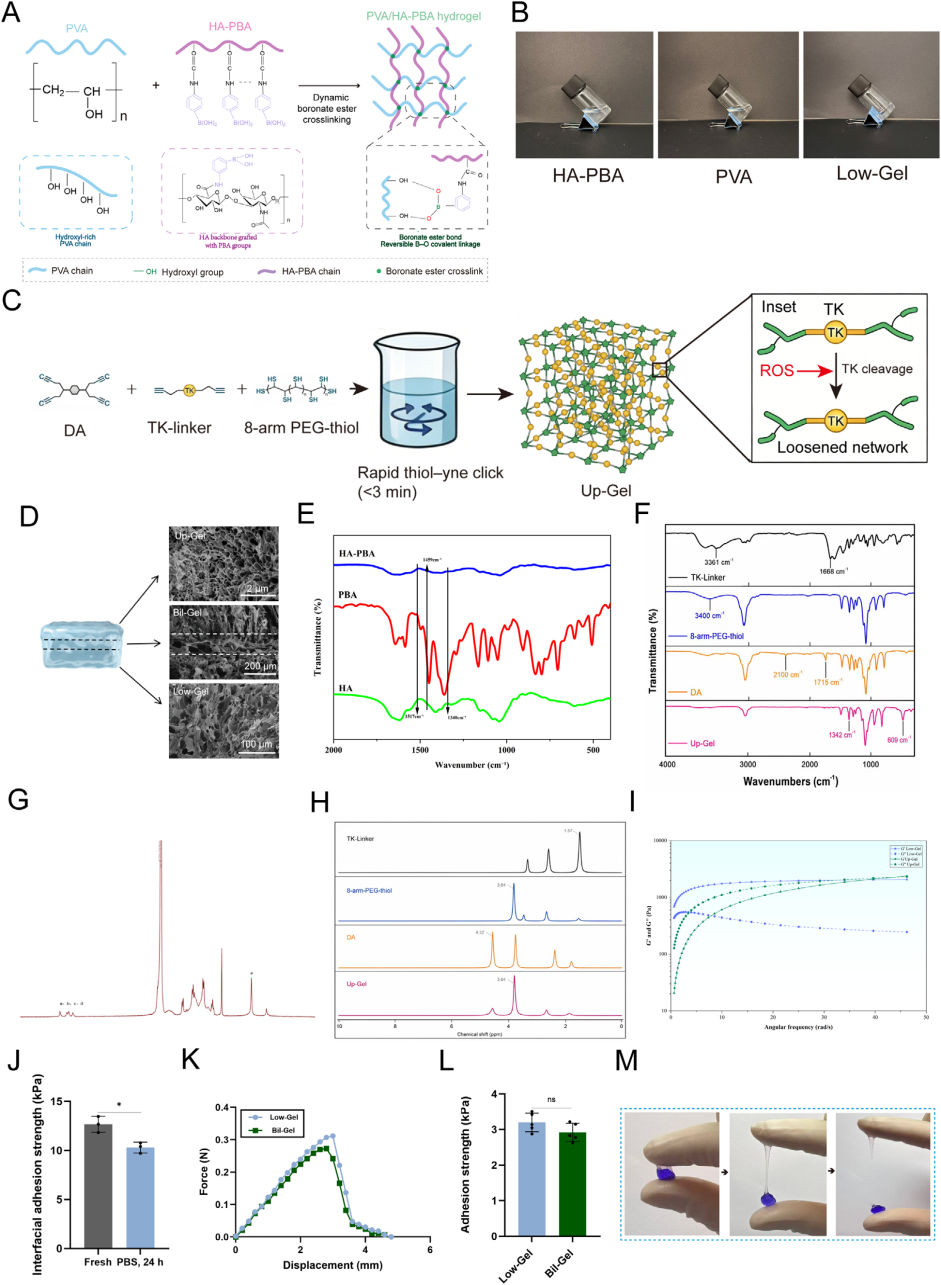
Fabrication and physicochemical characterization of the functionally compartmentalized bilayer hydrogel. (A) Reversible boronate‐ester crosslinking between HA‐PBA and PVA. (B) Representative vial‐inversion images showing rapid gelation of Low‐Gel after mixing HA‐PBA and PVA. (C) Formation of Up‐Gel through thiol–yne crosslinking and ROS‐responsive cleavage of thioketal junctions (inset: schematic illustration of ROS‐triggered thioketal cleavage and consequent network loosening). (D) Cross‐sectional SEM images of Up‐Gel, the Low‐Gel/Up‐Gel interface, and Low‐Gel (scale bars as indicated). (E) FTIR spectra of HA and HA‐PBA. (F) FTIR spectra of the Up‐Gel precursor components and the resulting Up‐Gel network. G) ^1H NMR spectra of HA and HA‐PBA. (H) ^1H NMR spectra of the Up‐Gel precursor components and the resulting Up‐Gel network. (I) Frequency‐sweep rheological analysis of the hydrogel compartments. (J) Interfacial adhesion strength of freshly prepared Bil‐Gel and Bil‐Gel after 24 h incubation in PBS at 37°C (n = 3 independent specimens per condition; two‐sided unpaired Student's t‐test). (K) Representative force–displacement curves for wet‐tissue adhesion. (L) Wet‐tissue adhesion strength of Low‐Gel and Bil‐Gel (n = 5 independent specimens per formulation; two‐sided unpaired Student's t‐test). (M) Representative macroscopic images of Bil‐Gel after manual stretching, showing cohesive failure away from the Low‐Gel/Up‐Gel interface. Quantitative data are presented as mean ± SD. ^*^ *p* < 0.05; ns, not significant. Schematic panels A and C were created using BioRender.com.

For Up‐Gel, poly(ethylene glycol) dipropiolate (PEG‐DA), a thioketal‐containing linker, and eight‐arm PEG‐thiol were crosslinked through thiol–yne chemistry. The resulting covalent PEG network incorporated oxidation‐sensitive thioketal junctions designed to cleave under elevated ROS conditions (Figure 2C). Relative to the precursor components, changes in the Fourier‐transform infrared spectroscopy (FTIR) andproton nuclear magnetic resonance (^1^H NMR) spectra were consistent with formation of the Up‐Gel network (Figure 2F,H). Up‐Gel was then formed directly on preformed Low‐Gel to produce an integrated bilayer while preserving spatial separation between the photodynamic and vesicle‐containing compartments.

Cross‐sectional SEM revealed porous structures within both layers together with continuous contact across the Low‐Gel/Up‐Gel interface, without an obvious interfacial gap in the examined sections (Figure 2D). Consistent with their different compositions and crosslinking modes, the two compartments also displayed distinct apparent pore‐size distributions (Figure S2A,B). Because the measurements were obtained from freeze‐dried specimens, these pore dimensions represent apparent morphological features rather than hydrated network mesh sizes.

The two compartments were further distinguished by their hydration and rheological behavior. In PBS at 37°C, the relative swelling ratio increased most strongly for Low‐Gel, followed by Up‐Gel, whereas Bil‐Gel exhibited less pronounced swelling (Figure S2C). Water uptake occurred primarily during the early incubation period and then gradually approached a plateau. During frequency‐sweep measurements, Up‐Gel remained predominantly elastic, with storage modulus consistently exceeding loss modulus, whereas Low‐Gel showed a stronger frequency‐dependent response (Figure 2I). These behaviors are consistent with the stable covalent network of Up‐Gel and the dynamic boronate‐ester network of Low‐Gel. After sectioning, Low‐Gel recovered upon reapposition of the freshly cut surfaces. Both Low‐Gel and Up‐Gel remained flexible and deformable during manual manipulation and could be molded into defined shapes, while Bil‐Gel maintained contact with representative solid substrates and freshly excised moist tissues (Figure S2F–K).

To determine whether the chemically distinct layers remained mechanically integrated after assembly, we examined interfacial integrity. Fresh Bil‐Gel showed an interfacial adhesion strength of 12.66 ± 0.82 kPa, which remained at 10.29 ± 0.55 kPa after 24 h in PBS at 37°C, corresponding to approximately 81% retention of the initial strength (Figure 2J). Rather than complete delamination along the Low‐Gel/Up‐Gel interface, failure occurred predominantly through mixed or bulk cohesive modes. In agreement with the quantitative measurements, manual stretching caused cohesive failure away from the interface instead of separation between the two layers (Figure 2M). Thus, despite the different network characteristics of Low‐Gel and Up‐Gel, the bilayer maintained mechanical continuity.

We separately assessed wet‐tissue adhesion to determine whether adding the upper compartment altered interaction of the wound‐contacting surface with moist tissue. Representative force–displacement profiles were similar for Low‐Gel and Bil‐Gel (Figure 2K). Adhesion strengths were 3.20 ± 0.26 and 2.92 ± 0.19 kPa, respectively, with no significant difference between the formulations (Figure 2L). Thus, addition of Up‐Gel did not measurably impair wet‐tissue adhesion provided by the Low‐Gel surface.

The two networks also exhibited different responses to oxidative conditions. With increasing H_2_O_2_ concentration, mass loss from Low‐Gel increased progressively, whereas Up‐Gel degraded more gradually despite accelerated degradation of its thioketal‐containing network under oxidative exposure (Figure S2D,E). These distinct degradation profiles are consistent with the different network chemistries and intended functions of the two layers. Low‐Gel localizes the Ce6‐containing photodynamic function at the wound interface, whereas the more persistent Up‐Gel provides a physically separated reservoir for engineered sEVs. Functional differentiation between the layers therefore arises from their combined spatial arrangement, network chemistry, and physicochemical behavior rather than from degradation kinetics alone.

Overall, the assembled Bil‐Gel preserved the distinct material properties of Low‐Gel and Up‐Gel together with interfacial integrity and wet‐tissue adhesion. The physical separation of the ROS‐generating photodynamic compartment from the engineered‐sEV reservoir, while maintaining mechanical continuity, provides a material basis for limiting direct overlap between the early oxidative antibacterial phase and subsequent vesicle delivery. This architecture forms the basis of the stage‐adaptive strategy evaluated in the following release and functional studies.

### 2.3 Temporal Release of Engineered sEV‐Associated Particles and Compatibility With Photodynamic Treatments

Using Up‐Gel@miR‐sEVs and Bil‐Gel@miR‐sEVs, we monitored release of engineered sEV‐associated particles over 14 days by NTA, with the bilayer formulation assessed both with and without photodynamic treatment (Figure 3A). Particle release from Up‐Gel@miR‐sEVs was relatively rapid, reaching approximately 78% cumulatively by day 3 and 93% by day 14. By contrast, Bil‐Gel@miR‐sEVs released only approximately 2% by day 3, after which release increased markedly between days 3 and 7 to reach approximately 92% cumulatively by day 14 (Figure 3B). Thus, bilayer assembly strongly restricted early particle release while exerting little effect on the final cumulative amount. This delayed profile is consistent with the additional transport barrier created by the bilayer architecture in combination with the distinct network and degradation properties of its two compartments.

**FIGURE 3:**
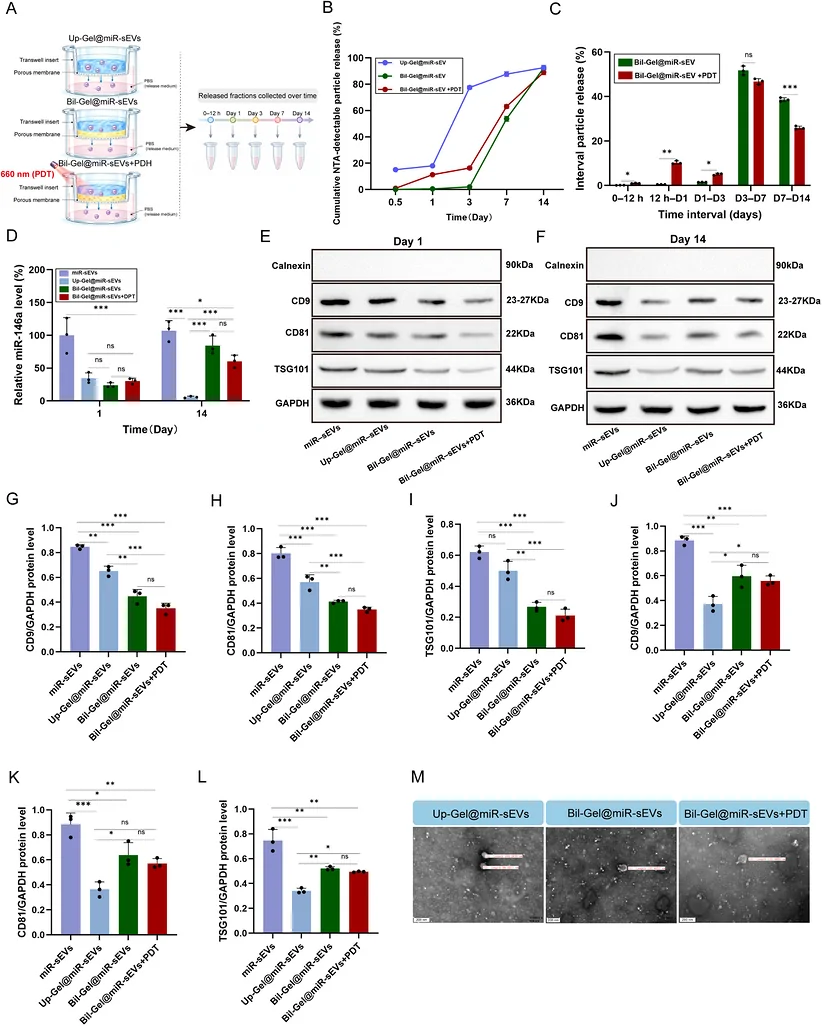
Temporal release of engineered sEV‐associated particles from the bilayer hydrogel and preservation of vesicle‐associated characteristics following photodynamic treatment. (A) Schematic of the Transwell‐based release assay. (B) Cumulative release of NTA‐detectable particles over 14 days. (C) Interval release of NTA‐detectable particles from Bil‐Gel@miR‐sEVs with or without photodynamic treatment. (D) Relative miR‐146a abundance in released fractions normalized to equal particle numbers on days 1 and 14, with the original miR‐sEV preparation used as the reference. (E, F) Representative western blots of Calnexin, CD9, CD81, TSG101, and GAPDH in released fractions collected on days 1 and 14. (G–I) Densitometric analysis of EV‐associated protein markers on day 1. (J–L) Densitometric analysis of EV‐associated protein markers on day 14. (M) Representative TEM images of vesicular particles recovered from the release fractions on day 7. Quantitative data are presented as mean ± SD (n = 3 independently prepared samples per condition). Cumulative and interval particle‐release profiles were analyzed by two‐way repeated‐measures ANOVA with Greenhouse–Geisser correction, followed by Šídák's multiple‐comparisons test. Data in D and G–L were analyzed by one‐way ANOVA followed by Tukey's post hoc test within each time point. ^*^ *p* < 0.05, ^**^ *p* < 0.01, ^***^ *p* < 0.001; ns, not significant. Schematic panel A was created using BioRender.com.

Photodynamic treatment changed the timing of particle release rather than its overall extent. Following irradiation, cumulative release from Bil‐Gel@miR‐sEVs reached approximately 16% by day 3 versus approximately 2% from the nonirradiated bilayer, while both groups approached 90% by day 14 (Figure 3B). Interval analysis revealed greater release after photodynamic therapy (PDT) during 0–12 h, 12 h–day 1, and day 1–3, no significant difference during day 3–7, and lower release during day 7–14 (Figure 3C). Thus, PDT redistributed a fraction of release toward earlier time points without increasing the final cumulative amount. Early release from the irradiated bilayer nevertheless remained substantially below that from Up‐Gel alone (approximately 16% versus 78% by day 3), indicating that a strong early‐release restriction was maintained by the bilayer under the applied photodynamic conditions.

To assess whether vesicle‐associated cargo was retained in the released fractions, we measured relative miR‐146a abundance after normalization to equal numbers of NTA‐detectable particles. On day 1, hydrogel‐derived fractions showed lower miR‐146a abundance than the original miR‐sEV preparation, whereas no significant differences were detected among Up‐Gel@miR‐sEVs and irradiated or nonirradiated Bil‐Gel@miR‐sEVs (Figure 3D). By day 14, relative miR‐146a abundance was higher in fractions recovered from the bilayer formulations than in those released from Up‐Gel alone. Irradiated and nonirradiated Bil‐Gel@miR‐sEVs remained statistically indistinguishable at both time points, indicating that the applied PDT regimen did not measurably decrease miR‐146a abundance within the released particle population.

We further examined the recovered fractions for EV‐associated protein markers. On days 1 and 14, CD9, CD81, and TSG101 remained detectable, whereas Calnexin was not detected (Figure 3E–L). Irradiated and nonirradiated Bil‐Gel@miR‐sEVs showed comparable marker profiles, with no evident loss of the major EV‐associated protein characteristics examined under the applied photodynamic conditions.

Complementary morphological evidence was obtained by TEM. Day‐7 fractions released from both Up‐Gel@miR‐sEVs and Bil‐Gel@miR‐sEVs contained nanoscale vesicular particles displaying rounded or cup‐shaped morphologies (Figure 3M). After PDT, similar morphologies were retained without obvious gross structural disruption under the tested conditions.

Overall, bilayer assembly substantially restricted early release of engineered sEV‐associated particles while still permitting extensive cumulative release over 14 days. Although PDT shifted part of this release toward earlier time points, it did not eliminate the delayed‐release behavior imposed by the bilayer. Following irradiation, the released fractions retained miR‐146a, major EV‐associated protein markers, and vesicular morphology. Together, these complementary measurements support preservation of key vesicle‐associated characteristics under the applied photodynamic regimen and are consistent with the intended temporal separation of the early photodynamic phase from subsequent vesicle delivery.

### 2.4 Complementary Redox Regulation and Cytoprotection

To determine whether the bilayer could accommodate the contrasting redox requirements of infected‐wound treatment, we further evaluated its compartment‐specific functions. Ce6‐containing Low‐Gel was positioned at the wound‐contacting surface to provide transient photooxidative activity during the early antibacterial phase, whereas the redox‐regulating Up‐Gel was designed to limit excessive oxidative stress during the subsequent repair period (Figure 4A). These opposing functions were therefore assigned to physically distinct compartments rather than co‐localized within a single hydrogel network.

**FIGURE 4:**
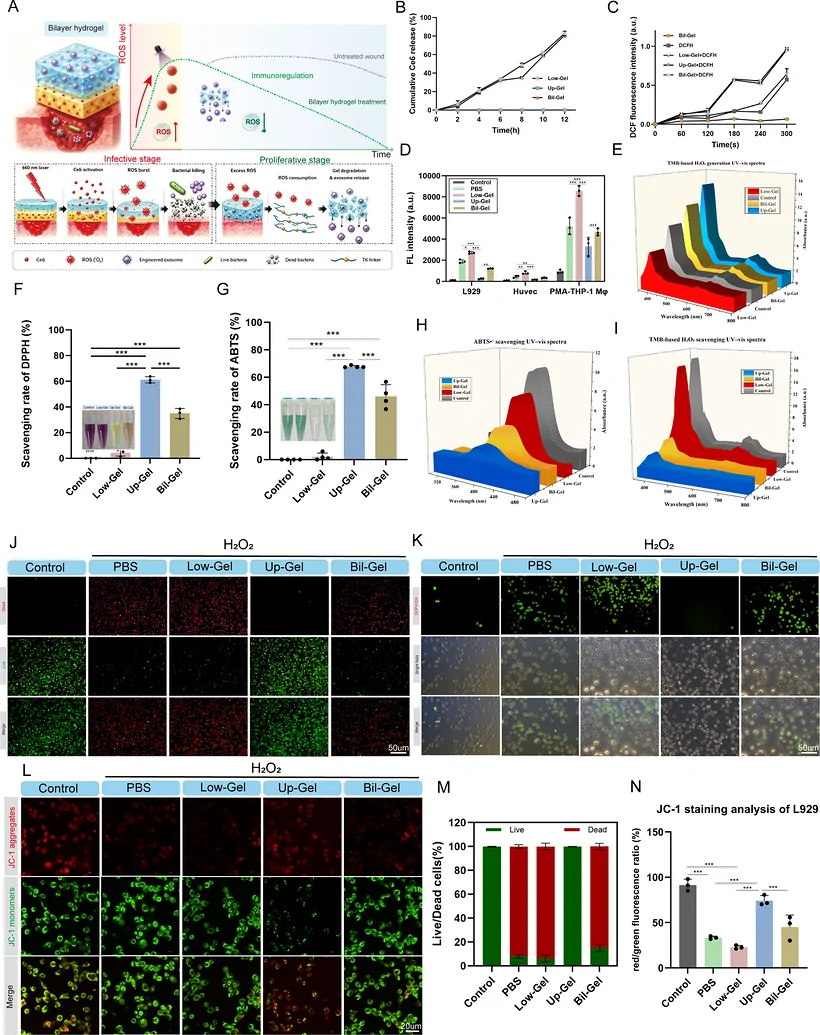
Complementary photooxidative and redox‐regulating functions of the bilayer hydrogel and associated cytoprotection. (A) Schematic illustration of the spatially compartmentalized redox functions of the bilayer hydrogel. (B) Cumulative release of chlorin e6 (Ce6) over 12 h. (C) Time‐dependent DCF fluorescence generated in the DCFH assay under 660 nm irradiation. (D) Quantification of intracellular DCF fluorescence in H_2_O_2_‐challenged L929 fibroblasts, HUVECs, and PMA‐differentiated THP‐1 macrophages. (E) Representative TMB‐based UV–vis spectra obtained under 660 nm irradiation. (F) DPPH radical‐scavenging activity. (G) ABTS radical‐scavenging activity. (H) Representative UV–vis spectra from the ABTS assay. (I) Representative TMB‐based UV–vis spectra following H_2_O_2_ exposure. (J) Representative live/dead staining images of L929 fibroblasts following H_2_O_2_ challenge (scale bar: 50 µm). (K) Representative intracellular DCF fluorescence images following DCFH‐DA staining and H_2_O_2_ challenge (scale bar: 50 µm). (L) Representative JC‐1 fluorescence images following H_2_O_2_ challenge (scale bar: 20 µm). (M) Quantification of L929 cell viability based on live/dead staining. (N) Quantification of the JC‐1 red‐to‐green fluorescence ratio. Quantitative data are presented as mean ± SD (n = 3 independent experiments). Data in B and C were analyzed by two‐way repeated‐measures ANOVA followed by Šídák's multiple‐comparisons test. Data in D, F, G, M, and N were analyzed by one‐way ANOVA followed by Tukey's post hoc test. Spectral data in E, H, and I are presented descriptively. ^*^ *p* < 0.05, ^**^ *p* < 0.01, ^***^ *p* < 0.001; ns, not significant. Schematic panel A was created using BioRender.com.

Ce6 release occurred predominantly within the early treatment window. Within the first 12 h, Low‐Gel and Bil‐Gel released most of their Ce6, reaching cumulative values of 82.76 ± 2.44% and 80.89 ± 1.84%, respectively (Figure 4B), whereas Up‐Gel showed no detectable Ce6 release. Bilayer assembly produced only a modest delay at intermediate time points, most clearly at 8 h, and by 12 h cumulative release from Bil‐Gel approached that from Low‐Gel. Thus, incorporation of Low‐Gel into the bilayer preserved the early Ce6 availability required for photodynamic treatment.

We assessed the photooxidative activity of released Ce6‐containing fractions using 2′,7′‐dichlorodihydrofluorescein (DCFH) and 3,3′,5,5′‐tetramethylbenzidine (TMB) assays. Under 660 nm irradiation, fractions from Low‐Gel and Bil‐Gel produced progressively greater probe oxidation, whereas only weak responses were observed for Up‐Gel (Figure 4C,E). Because these assays measure composite oxidative activity rather than individual ROS species, we interpreted them as complementary indicators of light‐induced oxidation. The comparable responses of Low‐Gel and Bil‐Gel show that incorporation into the bilayer did not materially impair the photoactive function of the wound‐contacting compartment.

We next examined the redox‐regulating properties of the upper compartment independently using cell‐free assays. Up‐Gel exhibited pronounced 2,2‐diphenyl‐1‐picrylhydrazyl (DPPH) radical‐scavenging activity, whereas the response of Low‐Gel was substantially weaker and that of Bil‐Gel was intermediate (Figure 4F). The same overall pattern was observed in the 2,2′‐azino‐bis(3‐ethylbenzothiazoline‐6‐sulfonic acid) (ABTS) measurements and corresponding ultraviolet–visible (UV–vis) spectra, with the strongest response from Up‐Gel and substantial activity retained by Bil‐Gel (Figure 4G,H). After H_2_O_2_ exposure, Up‐Gel also markedly decreased the TMB‐associated oxidative signal, while Bil‐Gel produced a smaller reduction (Figure 4I). Because this assay reflects the overall oxidative state of the reaction system rather than directly measuring residual H_2_O_2_, we interpreted it alongside the DPPH and ABTS measurements. Together, the concordant responses across these assays identify Up‐Gel as the principal redox‐regulating component of the bilayer.

We then examined the functional relevance of this redox‐regulating activity under cellular oxidative stress. H_2_O_2_ challenge markedly increased intracellular DCF fluorescence in L929 fibroblasts, HUVECs, and PMA‐differentiated THP‐1 macrophages. Across all three cell types, Up‐Gel substantially reduced the intracellular oxidative signal, while Bil‐Gel also produced a clear reduction (Figure 4D,K and Figure S3). At an equivalent total hydrogel amount, the effect of Bil‐Gel was generally weaker than that of Up‐Gel, consistent with the lower relative amount of the redox‐regulating upper component within the complete bilayer formulation.

The decrease in intracellular oxidative stress was accompanied by improved cell survival. In the PBS and Low‐Gel groups, H_2_O_2_ exposure caused extensive loss of viable L929 cells, whereas Up‐Gel markedly preserved cell viability and Bil‐Gel maintained substantial cytoprotective activity (Figure 4J,M). A parallel pattern was observed for mitochondrial membrane potential. H_2_O_2_ markedly reduced the JC‐1 red‐to‐green fluorescence ratio, consistent with mitochondrial depolarization; Up‐Gel substantially restored this ratio, whereas Bil‐Gel produced a clear but partial recovery (Figure 4L,N). Thus, redox regulation by the upper compartment was associated with reduced intracellular oxidative stress, preservation of cell viability, and attenuation of oxidative mitochondrial injury.

Within the bilayer, the two compartments therefore provided complementary rather than redundant redox functions. Low‐Gel preserved the transient photooxidative activity needed for early photodynamic treatment, whereas Up‐Gel moderated excessive oxidative stress and protected cells involved in inflammatory regulation and tissue repair. By spatially separating these opposing functions, the bilayer provides a physicochemical basis for coupling an early ROS‐dependent antibacterial phase to a subsequent microenvironment more favorable for engineered‐sEV‐mediated immunoregulation and tissue regeneration.

### 2.5 Photodynamic Antibacterial Activity, Inhibition of Biofilm Formation, and Activity Against Pre‐Established Biofilms

We examined the antibacterial activity of the Ce6‐containing wound‐contacting compartment against *Staphylococcus aureus*, *Escherichia coli*, and a multidrug‐resistant *Pseudomonas aeruginosa* (MDR *P. aeruginosa*) strain (Figure 5A). In the absence of irradiation, the hydrogel formulations had little effect on recoverable bacterial colony‐forming units (CFUs). After 660 nm irradiation, however, both Low‐Gel and Bil‐Gel markedly reduced bacterial recovery across all three species (Figure 5C,G). The comparable responses of these formulations indicate that addition of the upper compartment did not appreciably impair the photodynamic antibacterial function of the Ce6‐containing lower layer.

**FIGURE 5:**
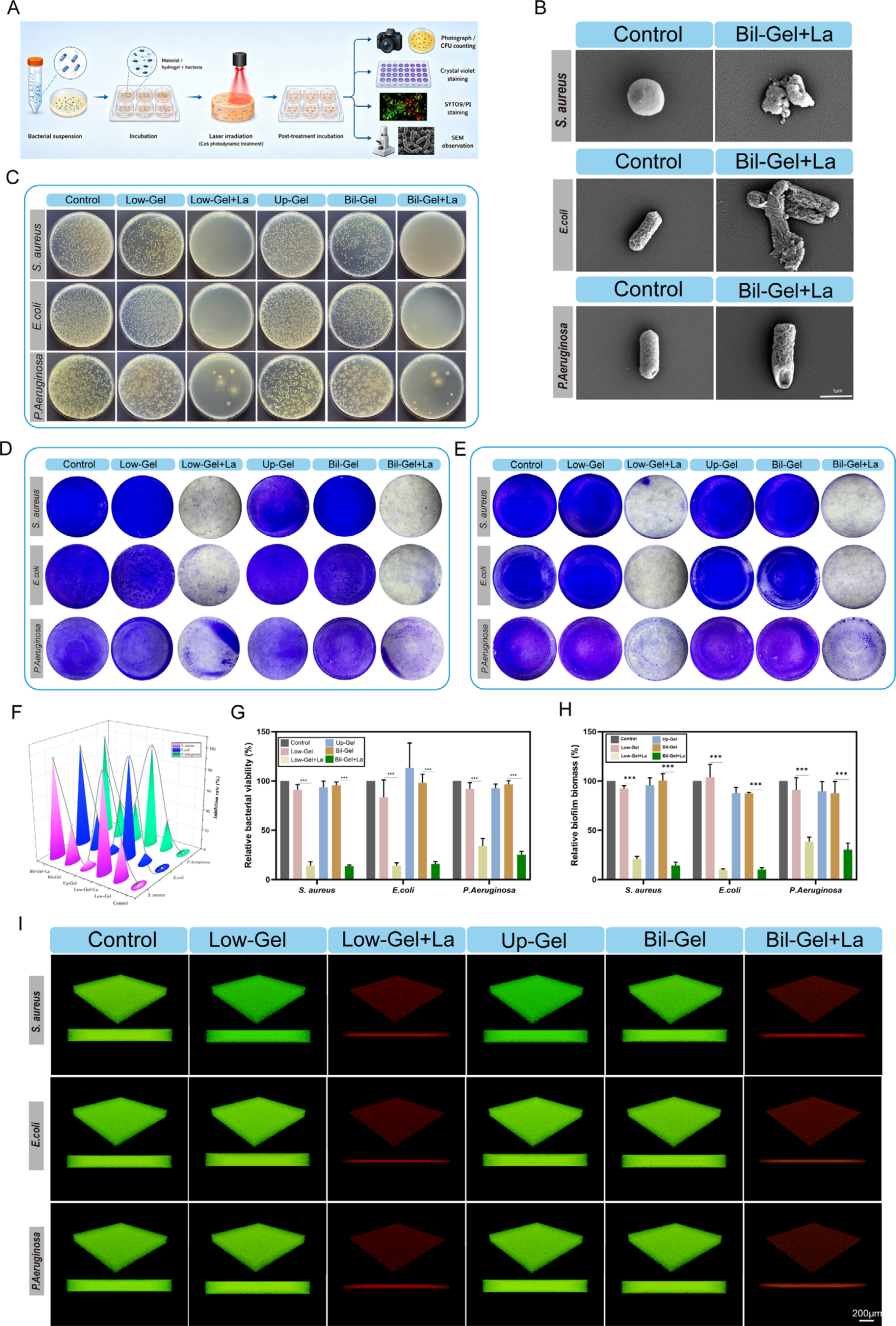
Light‐triggered antibacterial activity, inhibition of biofilm formation, and activity against pre‐established biofilms of the bilayer hydrogel. (A) Schematic illustration of the experimental workflow. (B) Representative grayscale SEM images of Staphylococcus aureus, Escherichia coli, and MDR Pseudomonas aeruginosa in the control and Bil‐Gel+La groups (scale bars: 1 µm). (C) Representative bacterial colony‐forming plates under the indicated treatment conditions. (D) Representative crystal violet staining images of biofilms formed under the indicated treatment conditions. (E) Representative crystal violet staining images of pre‐established 48 h biofilms after treatment. (F) Quantification of biofilm formation based on crystal violet staining. (G) Relative bacterial survival based on CFU recovery. (H) Quantification of residual biomass in pre‐established biofilms. (I) Representative three‐dimensional SYTO9/PI fluorescence images showing biofilm architecture and membrane‐integrity‐associated fluorescence (scale bars: 200 µm). “+La” denotes 660 nm laser irradiation. Quantitative data are presented as mean ± SD (n = 3 independent experiments). Statistical comparisons were performed separately for each bacterial species using one‐way ANOVA followed by Tukey's post hoc test. ^*^ *p* < 0.05, ^**^ *p* < 0.01, ^***^ *p* < 0.001; ns, not significant. Schematic panel A was created using BioRender.com.

Following photodynamic treatment, scanning electron microscopy (SEM) revealed substantial morphological disruption. Untreated *S. aureus* maintained its typical coccoid morphology, while *E. coli* and *P. aeruginosa* retained intact rod‐shaped structures. By contrast, bacteria exposed to irradiated Bil‐Gel displayed irregular, deformed surfaces accompanied by varying degrees of aggregation and structural collapse (Figure 5B). These morphological alterations were consistent with the observed reduction in recoverable CFUs.

We next used crystal violet staining to assess biofilm formation. In the control and nonirradiated groups, dense surface‐associated biomass was retained, whereas irradiation of Low‐Gel or Bil‐Gel substantially decreased crystal violet‐positive biomass across all three bacterial species (Figure 5D,F). Thus, after incorporation into the bilayer construct, the Ce6‐containing compartment retained its capacity to suppress biofilm accumulation.

Because suppression of biofilm formation does not necessarily indicate activity against established biofilms, we subsequently tested 48 h pre‐established biofilms under the same treatment conditions. Substantial attached biomass persisted in the untreated and nonirradiated groups, whereas irradiation of Low‐Gel or Bil‐Gel markedly reduced the residual biofilm biomass (Figure 5E,H). Thus, the photodynamic effect remained evident after biofilm establishment rather than being limited to interference with early biofilm development.

To complement these measurements, three‐dimensional SYTO9/PI imaging was used to evaluate membrane integrity and biofilm architecture. Biofilms in the control and nonirradiated groups were dominated by SYTO9‐associated fluorescence and maintained relatively dense three‐dimensional structures. After photodynamic treatment with Low‐Gel or Bil‐Gel, SYTO9‐associated fluorescence decreased while propidium iodide (PI)‐associated fluorescence increased, and the remaining biofilm structures were thinner and less continuous (Figure 5I). This pattern was consistent with increased bacterial membrane damage accompanied by disruption of biofilm architecture.

Together, these results show that the Ce6‐containing lower compartment retained robust light‐dependent antibacterial activity against all three tested bacterial species while reducing both developing and 48 h pre‐established biofilms. Importantly, incorporation into Bil‐Gel preserved these effects, indicating that the upper compartment did not compromise the early photodynamic antibacterial function of the wound‐contacting layer.

### 2.6 miR‐146a‐Enriched Engineered sEVs Attenuate IRAK1/NF‐κB Signaling in Human Macrophages with Partial CD64 Dependence

Following the experimental workflow shown in Figure 6A, we examined the immunoregulatory activity of engineered miR‐sEVs in PMA‐differentiated THP‐1 macrophages. Relative to undifferentiated THP‐1 cells, PMA differentiation increased the proportion of CD64‐positive cells (Figure S4), confirming expression of the intended receptor in this human macrophage model and establishing an appropriate setting for assessing the functional contribution of the incorporated human CD64‐recognition module.

**FIGURE 6:**
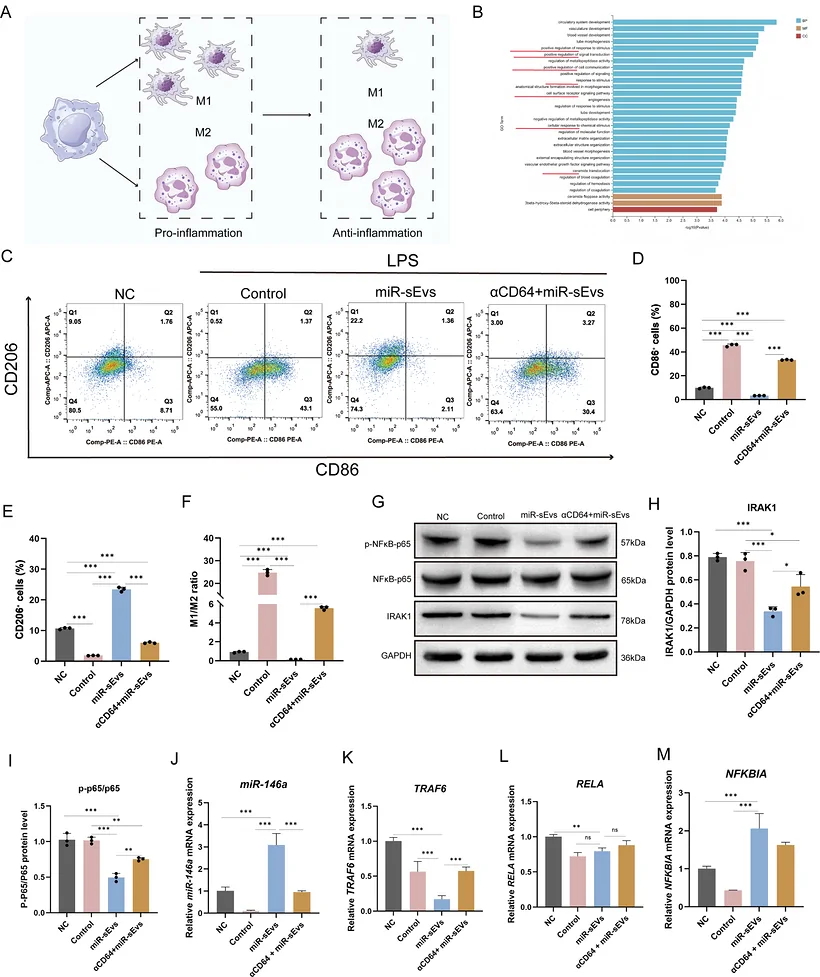
miR‐146a‐enriched engineered sEVs attenuate IRAK1/NF‐κB signaling and promote a repair‐associated macrophage phenotype with partial CD64 dependence. (A) Schematic of the experimental workflow and proposed immunoregulatory mechanism. (B) Gene Ontology (GO) enrichment analysis of differentially expressed genes following miR‐sEV treatment. (C) Representative flow‐cytometry plots of CD86 and CD206 expression. (D–F) Quantification of CD86‐positive cells, CD206‐positive cells, and the CD86‐positive/CD206‐positive ratio, respectively. (G) Representative western blots of IRAK1, phosphorylated NF‐κB p65 (p‐p65), total p65, and GAPDH. (H, I) Quantification of IRAK1/GAPDH and p‐p65/total p65 ratios, respectively. (J–M) qRT‐PCR analysis of miR‐146a, TRAF6, RELA, and NFKBIA expression. Statistical comparisons for qRT‐PCR data were performed using ΔCt values, while relative expression values are shown for visualization. Quantitative data are presented as mean ± SD (n = 3 independent experiments). Data in D–F, H–I, and J–M were analyzed by one‐way ANOVA followed by Tukey's post hoc test. ^*^ *p* < 0.05, ^**^ *p* < 0.01, ^***^ *p* < 0.001; ns, not significant. Schematic panel A was created using BioRender.com.

We first used transcriptomic profiling to assess the macrophage response to miR‐sEV treatment. Relative to vehicle‐treated LPS‐stimulated macrophages, treatment with miR‐sEVs produced 119 upregulated and 103 downregulated genes (Figure S5A,B), indicating a broader transcriptional response to the engineered vesicles. Gene Ontology (GO) enrichment analysis identified terms associated with inflammatory and immune regulation together with processes related to vascular development and tissue repair (Figure 6B). Complementary Kyoto Encyclopedia of Genes and Genomes (KEGG) pathway over‐representation analysis was also performed for the screened genes (Figure S5E). Changes in genes associated with inflammatory regulation and macrophage function were also evident in a focused heatmap (Figure S5D). Thus, the transcriptomic response suggests that miR‐sEV treatment may affect inflammatory and repair‐related processes beyond individual inflammatory mediators. Gene set enrichment analysis (GSEA) additionally showed a trend toward enrichment of the myeloid leukocyte differentiation gene set (Figure S5C). Although this enrichment was no longer statistically significant after multiple‐testing correction, its direction was broadly consistent with the macrophage functional changes indicated by the other transcriptomic analyses.

A corresponding shift in macrophage surface‐marker profiles was observed by flow cytometry. Relative to unstimulated controls, LPS stimulation increased the proportion of CD86‐positive cells while reducing CD206 positivity. These LPS‐induced changes were attenuated by miR‐sEV treatment, which resulted in lower CD86 positivity, higher CD206 positivity, and a reduced CD86‐positive/CD206‐positive cell ratio (Figure 6C–F). This surface‐marker pattern was consistent with attenuation of the LPS‐induced inflammatory macrophage phenotype.

We next evaluated the contribution of CD64 recognition through receptor blockade. Pretreatment with an anti‐CD64 antibody partially attenuated the effects of miR‐sEVs on macrophage surface‐marker expression. Compared with miR‐sEV treatment alone, CD64 blockade increased CD86 positivity and decreased CD206 positivity (Figure 6C–F). Because the reversal was incomplete, these results indicate that CD64 contributes to the macrophage response to the engineered vesicles while additional vesicle–cell interaction or uptake pathways remain operative.

The molecular response followed the phenotypic changes. Treatment with miR‐sEVs increased intracellular miR‐146a abundance (Figure 6J) while reducing IRAK1 protein abundance (Figure 6G,H). NF‐κB p65 phosphorylation also decreased, whereas total p65 abundance remained comparatively stable (Figure 6G,I). At the transcriptional level, *TRAF6* expression decreased, *NFKBIA* expression increased, and *RELA* showed only modest variation (Figure 6K–M). Together, this expression pattern was consistent with attenuation of IRAK1‐associated NF‐κB signaling after delivery of miR‐146a‐enriched engineered sEVs.

Following CD64 blockade, intracellular miR‐146a accumulation was partially reduced, and miR‐sEV‐induced suppression of IRAK1 expression and p65 phosphorylation was weakened. The corresponding changes in intracellular miR‐146a, IRAK1/NF‐κB signaling, and CD86/CD206 profiles support a functional link between engineered‐sEV delivery and attenuation of inflammatory signaling in macrophages. Because these responses were only partially reversed by receptor blockade, CD64 appears to facilitate vesicle uptake and downstream immunoregulatory activity rather than determine them exclusively.

Previous studies have established miR‐146a as an important negative regulator of innate inflammatory signaling through targets including IRAK1 and TRAF6 [25, 26], while EV‐mediated delivery of miR‐146a has been investigated in inflammatory and diabetic wound settings [27, 28, 29]. Here, combining miR‐146a enrichment with a human CD64‐recognition module allowed the contribution of receptor recognition to be examined directly. The concordance among the transcriptomic response, macrophage surface‐marker changes, increased intracellular miR‐146a, attenuation of IRAK1/NF‐κB signaling, and partial loss of these effects after CD64 blockade links the molecular design of miR‐sEVs to their immunoregulatory activity. Together, these findings support a CD64‐assisted, but not CD64‐exclusive, interaction between the engineered vesicles and human macrophages and provide a biological rationale for incorporating them into the upper hydrogel compartment to locally modulate the inflammatory wound microenvironment.

### 2.7 Engineered miR‐sEVs Retain Repair‐Associated Activity Following Incorporation into the Upper Hydrogel Compartment

We evaluated the repair‐associated activity of engineered miR‐sEVs in human umbilical vein endothelial cells (HUVECs) and L929 fibroblasts. Free miR‐sEVs at an equivalent nominal dose were used as a reference, while the corresponding blank hydrogel formulations were assessed in parallel to distinguish vesicle‐associated responses from those attributable to the hydrogel matrices.

In HUVECs, free miR‐sEVs markedly increased 5‐ethynyl‐2′‐deoxyuridine (EdU) incorporation, and a clear proliferative response was retained after vesicle incorporation into Up‐Gel (Figure 7A,F). Free miR‐sEVs also accelerated scratch closure at 12 and 24 h and enhanced Transwell migration, whereas Up‐Gel@miR‐sEVs produced responses in the same direction but at lower magnitude during the short assay period (Figure 7B,E,G,K). Consistent with the restricted early vesicle availability demonstrated in the release experiments, Bil‐Gel@miR‐sEVs elicited a more modest early response.

**FIGURE 7:**
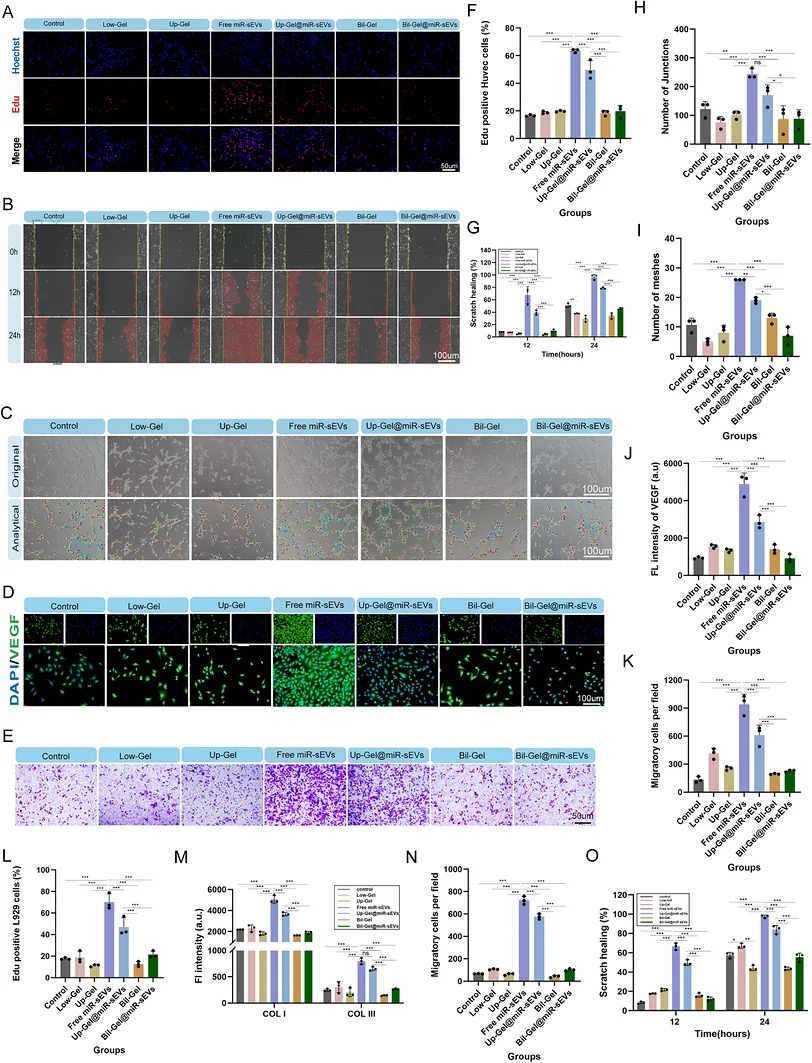
Engineered miR‐sEVs retain repair‐associated activity in endothelial cells and fibroblasts after incorporation into the upper hydrogel compartment. (A–E) Representative images of EdU staining, scratch‐wound assay, tube formation, VEGF immunofluorescence, and Transwell migration in HUVECs, respectively (scale bars as indicated). (F–K) Corresponding quantitative analyses of HUVEC proliferation, migration, tube formation, and VEGF expression. (L–O) Quantification of EdU incorporation, collagen I/III fluorescence, Transwell migration, and scratch closure in L929 fibroblasts. Quantitative data are presented as mean ± SD (n = 3 independent biological replicates). Data in F and H–N were analyzed by one‐way ANOVA followed by Tukey's post hoc test. Time‐dependent data in G and O were analyzed by two‐way repeated‐measures ANOVA followed by Šídák's multiple‐comparisons test. ^*^ *p* < 0.05, ^**^ *p* < 0.01, ^***^ *p* < 0.001; ns, not significant.

A similar relationship among formulations was observed for endothelial network formation. Both junction and mesh numbers increased with free miR‐sEVs, while substantial network‐forming activity was retained after incorporation into Up‐Gel (Figure 7C,H,I). Free miR‐sEVs also increased vascular endothelial growth factor (VEGF)‐associated fluorescence, which remained elevated in the Up‐Gel@miR‐sEVs group relative to the control and blank hydrogel groups (Figure 7D,J). Over the same experimental interval, Bil‐Gel@miR‐sEVs again produced a weaker response, consistent with the delayed early release imposed by the complete bilayer architecture.

We further examined the activity of the engineered vesicles in L929 fibroblasts. Free miR‐sEVs increased EdU incorporation, Transwell migration, and scratch closure, with these responses remaining evident after the vesicles were incorporated into Up‐Gel (Figure 7L,N,O and Figure S6A–D). Following miR‐sEV treatment, collagen I‐ and collagen III‐associated fluorescence also increased and remained elevated in the Up‐Gel@miR‐sEVs group (Figure 7M and Figure S6). Within the short experimental window, Bil‐Gel@miR‐sEVs produced less pronounced responses, paralleling the pattern observed in HUVECs.

Across endothelial cells and fibroblasts, these responses show that incorporation into Up‐Gel preserved substantial repair‐associated activity of the engineered vesicles. Free miR‐sEVs generally produced the strongest short‐term effects, whereas Bil‐Gel@miR‐sEVs elicited a more restrained early response. This hierarchy closely paralleled the independently determined release profiles in Figure 3, in which vesicle‐associated particles became available earlier from Up‐Gel while early release was markedly restricted by the complete bilayer. The comparatively modest short‐term effects of Bil‐Gel@miR‐sEVs are therefore consistent with its temporally regulated vesicle availability rather than indicating an evident loss of repair‐associated activity after hydrogel incorporation.

### 2.8 Bil‐Gel@miR‐sEVs Coordinates Infection Control and Tissue Repair in Infected Diabetic Wounds

To evaluate bilayer hydrogel performance in vivo, we established a polymicrobial full‐thickness wound model in diabetic mice using an equal‐volume mixture of *Staphylococcus aureus*, *Escherichia coli*, and MDR *Pseudomonas aeruginosa* (1:1:1, v/v/v) (Figure 8A). Animals were assigned to six groups: I, infected control; II, Low‐Gel; III, Up‐Gel@miR‐sEVs; IV, Bil‐Gel; V, Bil‐Gel@sEVs; and VI, Bil‐Gel@miR‐sEVs. Following irradiation of Ce6‐containing formulations at 660 nm according to the predefined protocol, wound healing was monitored for 14 days (Figure 8B–D).

**FIGURE 8:**
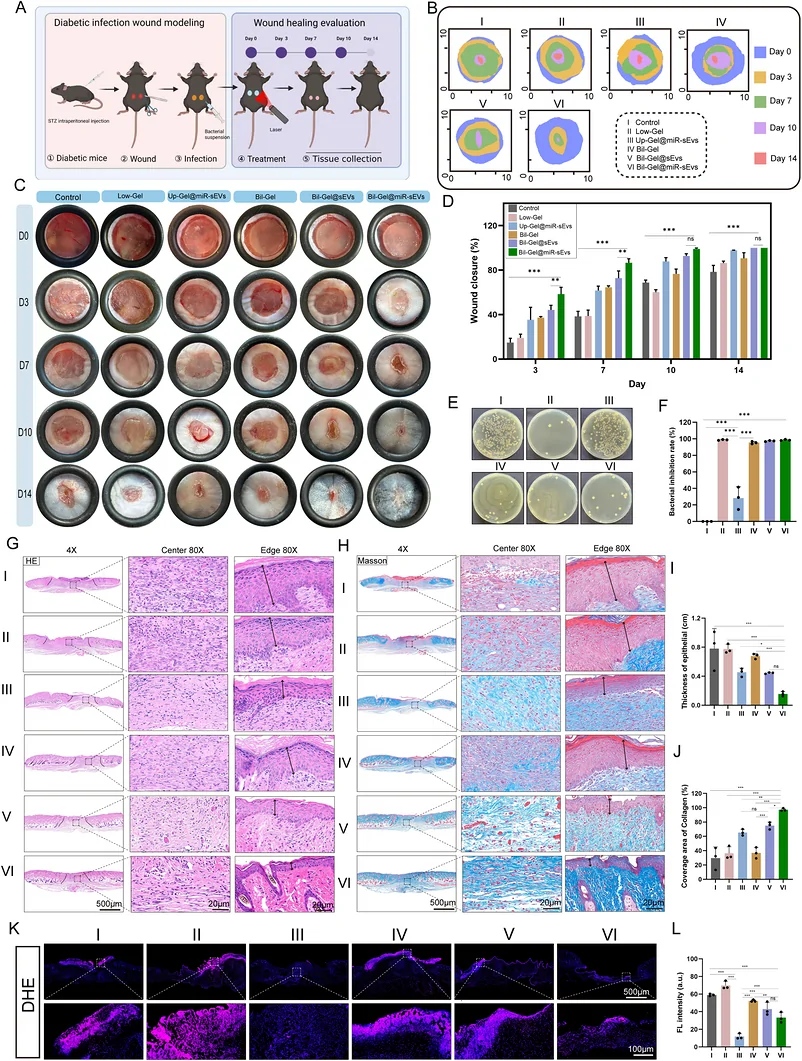
Bil‐Gel@miR‐sEVs coordinates infection control and tissue repair in infected diabetic wounds. (A) Schematic of the polymicrobial infected diabetic wound model established using an equal‐volume mixture of *S. aureus*, *E. coli*, and MDR *P. aeruginosa* (1:1:1, v/v/v), together with the treatment and sampling schedule. (B, C) Wound‐area tracings and representative wound photographs during the 14‐day treatment period. (D) Quantification of wound closure over 14 days. (E, F) Representative bacterial colony plates and corresponding bacterial inhibition rates on day 3. (G, H) Representative H&E‐ and Masson's trichrome‐stained wound sections on day 14 (scale bars: 500, 20, and 20 µm, as indicated). (I, J) Quantification of neoepidermal thickness and collagen‐positive area on day 14. (K, L) Representative DHE‐stained wound sections and quantification of DHE‐associated fluorescence intensity on day 3 (scale bars: 500 and 100 µm, as indicated). Quantitative data are presented as mean ± SD (n = 3 independent mice per quantified endpoint). Independent cohorts were used for individual endpoints as described in the Experimental Section. Data in D were analyzed by two‐way repeated‐measures ANOVA followed by Šídák's multiple‐comparisons test. Data in F, I, J, and L were analyzed by one‐way ANOVA followed by Tukey's post hoc test. ^*^ *p* < 0.05, ^**^ *p* < 0.01, ^***^ *p* < 0.001; ns, not significant. Schematic panel A was created using BioRender.com.

Although wound closure progressed in all treatment groups, both its rate and extent differed substantially among formulations. Relative to the infected control, Low‐Gel accelerated wound resolution, consistent with a contribution from early photodynamic infection control. Healing was also improved by Up‐Gel@miR‐sEVs, although without comparable early antibacterial activity. Combining the two compartments further accelerated wound closure, and Bil‐Gel@sEVs outperformed the corresponding single‐component treatments. The most favorable overall healing course was observed with Bil‐Gel@miR‐sEVs, which approached complete closure by day 14 (Figure 8B–D). Thus, the therapeutic outcome achieved with the complete bilayer formulation was not reproduced by either early antibacterial treatment or vesicle‐mediated repair alone.

To assess the early antibacterial contribution in vivo, we measured wound bacterial burden on day 3. Recoverable bacteria were markedly reduced by Low‐Gel and both Ce6‐containing bilayer formulations, whereas Up‐Gel@miR‐sEVs produced only limited antibacterial activity (Figure 8E,F). Consistent with the in vitro findings, these results indicate that after bilayer assembly, the wound‐contacting Low‐Gel compartment remained the principal contributor to early photodynamic bacterial clearance. Addition of the upper sEV‐containing compartment therefore did not diminish this antibacterial function.

We evaluated the early oxidative state of the wound by dihydroethidium (DHE) staining on day 3. Strong fluorescence was observed in infected control wounds, indicating pronounced oxidative stress. Despite effectively reducing bacterial burden, photodynamic treatment with Low‐Gel alone did not fully alleviate the persistent oxidative environment. In contrast, DHE fluorescence was lower with the bilayer formulations and showed the greatest reduction in the Bil‐Gel@miR‐sEVs group (Figure 8K,L). This pattern reflects the functional organization of the bilayer system, in which the lower compartment provides an early ROS‐dependent photodynamic effect at the wound interface while the upper compartment contributes to subsequent control of excessive oxidative stress.

By day 14, histological analysis revealed clear differences in tissue reconstruction. Infected control wounds remained incompletely epithelialized, with disorganized granulation tissue and limited collagen deposition. Following infection control, Low‐Gel improved tissue organization, whereas Up‐Gel@miR‐sEVs promoted regenerative features without a comparable early antibacterial effect. Bilayer treatment produced more continuous neoepithelial coverage and better‐organized collagen deposition, with the most advanced histological repair observed for Bil‐Gel@miR‐sEVs (Figure 8G–J). Hematoxylin and eosin (H&E) and Masson's trichrome staining therefore supported a more effective transition from early infection control to subsequent tissue reconstruction in this group.

Overall, Bil‐Gel@miR‐sEVs exhibited the most favorable therapeutic profile among the tested formulations. Its treatment course combined rapid Ce6‐mediated bacterial control during the early phase with subsequent improvement of the wound microenvironment and tissue reconstruction during repair. Bil‐Gel@sEVs also enhanced healing, whereas the additional benefit of Bil‐Gel@miR‐sEVs suggests a contribution from the engineered vesicle cargo. These findings are consistent with the stage‐adaptive bilayer design, in which spatially separated therapeutic compartments address the changing requirements of infected diabetic wound healing.

### 2.9 Bil‐Gel@miR‐sEVs Attenuates IRAK1/NF‐κB Signaling and Supports Reparative Remodeling In Vivo

To examine the biological changes associated with improved wound repair after early infection control, we assessed inflammatory signaling and macrophage‐associated tissue markers on day 7, followed by vascularization, cellular proliferation, and tissue remodeling on day 14. On day 7, IRAK1, phosphorylated NF‐κB p65 (p‐p65), and interleukin‐6 (IL‐6) signals remained prominent in the infected control and Low‐Gel groups but were reduced in the sEV‐containing formulations, with the lowest levels observed for Bil‐Gel@miR‐sEVs among the evaluated groups (Figure 9A–C,G). These findings associate the sEV‐containing upper compartment with attenuation of the inflammatory wound environment after the early antibacterial phase.

**FIGURE 9:**
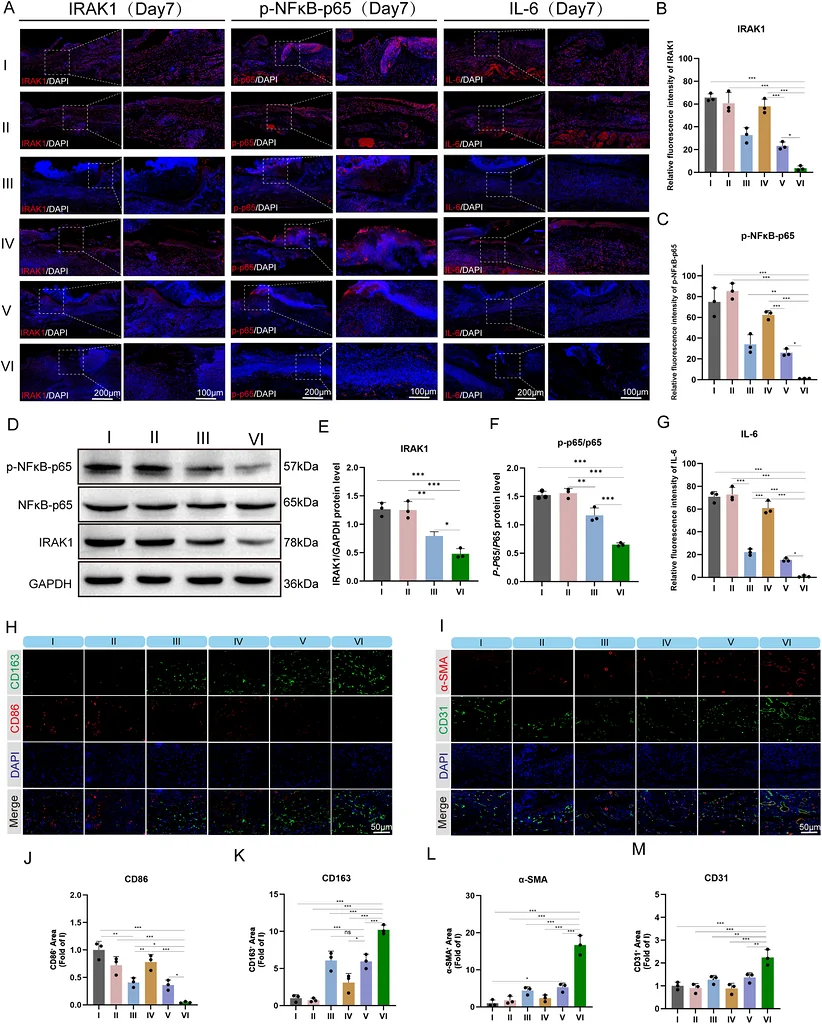
Bil‐Gel@miR‐sEVs attenuates IRAK1/NF‐κB signaling and promotes reparative remodeling in vivo. (A) Representative immunofluorescence images of IRAK1, phosphorylated NF‐κB p65 (p‐p65), and IL‐6 in wound tissues on day 7. (B, C) Quantification of IRAK1 and p‐p65 fluorescence intensity, respectively. (D–F) Representative Western blots and densitometric quantification of IRAK1/GAPDH and the p‐p65/total p65 ratio in the infected control, Low‐Gel, Up‐Gel@miR‐sEVs, and Bil‐Gel@miR‐sEVs groups. (G) Quantification of IL‐6 fluorescence intensity on day 7. (H) Representative CD163/CD86 immunofluorescence images in wound tissues on day 7. (I) Representative α‐SMA/CD31 immunofluorescence images in wound tissues on day 14. (J, K) Quantification of CD86‐ and CD163‐positive areas, respectively. (L, M) Quantification of α‐SMA‐ and CD31‐positive areas, respectively. Scale bars are indicated in the corresponding panels. Quantitative data are presented as mean ± SD (n = 3 independent animals per group). Statistical comparisons were performed using one‐way ANOVA followed by Tukey's post hoc test. ^*^ *p* < 0.05, ^**^ *p* < 0.01, ^***^ *p* < 0.001; ns, not significant.

Western blotting was conducted in four representative groups: infected control, Low‐Gel, Up‐Gel@miR‐sEVs, and Bil‐Gel@miR‐sEVs. Relative to the infected control and Low‐Gel groups, Bil‐Gel@miR‐sEVs reduced IRAK1 protein abundance and the p‐p65/total p65 ratio (Figure 9D–F). This pattern was consistent with the attenuation of IRAK1/NF‐κB signaling observed in the in vitro macrophage experiments and supports involvement of miR‐146a‐associated regulation within the wound environment. The agreement between cell‐ and tissue‐level findings provides complementary evidence that attenuation of IRAK1/NF‐κB signaling contributes to the immunoregulatory activity of Bil‐Gel@miR‐sEVs in vivo.

Changes in macrophage‐associated tissue markers paralleled the reduction in inflammatory signaling. Extensive CD86‐positive staining with relatively limited CD163‐positive staining was observed in the infected control and Low‐Gel groups, whereas the sEV‐containing formulations showed smaller CD86‐positive areas and larger CD163‐positive areas. This reciprocal shift was most pronounced in the Bil‐Gel@miR‐sEVs group (Figure 9H,J,K). The pattern is consistent with a transition from a predominantly inflammatory macrophage‐associated state toward a more repair‐associated tissue environment and accords with the macrophage response observed in vitro. Similar regulation of the inflammatory wound microenvironment has been implicated in the reparative effects of immunomodulatory hydrogel systems [49, 50, 51].

Representative H&E‐ and Masson's trichrome‐stained wound sections on day 7 showed the tissue morphology at this intermediate stage of healing (Figure S7A,B). These day‐7 responses followed the early antibacterial effects observed on day 3. The wound‐contacting lower compartment rapidly reduced bacterial burden through Ce6‐mediated photodynamic treatment, whereas attenuation of IRAK1/NF‐κB‐associated signaling and changes in macrophage‐associated tissue markers became evident at the later time point. This sequence is consistent with the intended functional organization of the bilayer system, in which early infection control precedes regulation of the inflammatory wound microenvironment.

By day 14, the tissue response had shifted further toward reparative remodeling. Relative to the infected control and non‐sEV hydrogel groups, Bil‐Gel@miR‐sEVs increased the abundance of CD31‐positive vascular profiles and alpha‐smooth muscle actin (α‐SMA)‐positive structures (Figure 9I,L,M). Complementary immunofluorescence analysis showed greater VEGF‐associated fluorescence and increased Ki67‐associated fluorescence intensity in the Bil‐Gel@miR‐sEVs group. (Figure S7C–F). Concurrent increases in CD31 and VEGF were consistent with enhanced vascular reconstruction, whereas increased Ki67‐associated fluorescence was consistent with greater proliferative activity within the healing tissue. Increased α‐SMA staining further supported ongoing vascular‐associated and stromal remodeling during the later repair phase.

Across the day‐3, day‐7, and day‐14 assessments, the wound response progressed from early bacterial control to attenuation of inflammatory signaling and changes in macrophage‐associated phenotype, followed by vascular and tissue reconstruction. This temporal progression is consistent with the stage‐adaptive design of Bil‐Gel@miR‐sEVs: the wound‐contacting lower compartment provides early photodynamic infection control, while the upper engineered‐sEV‐containing compartment contributes to subsequent regulation of the wound microenvironment and reparative remodeling. The in vivo findings therefore support a temporally coordinated therapeutic response across distinct stages of infected diabetic wound healing.

### 2.10 Day‐14 Transcriptomics Reveals a Shift from Inflammatory–Myeloid Programs Toward Epithelial and Matrix‐Remodeling Signatures

We performed bulk RNA sequencing on whole‐wound tissues collected on day 14 from infected control and Bil‐Gel@miR‐sEVs‐treated mice, using three independent biological samples per group. Following Bil‐Gel@miR‐sEVs treatment, differential‐expression analysis identified 341 significantly altered genes, comprising 87 upregulated and 254 downregulated genes (Figure 10A).

**FIGURE 10:**
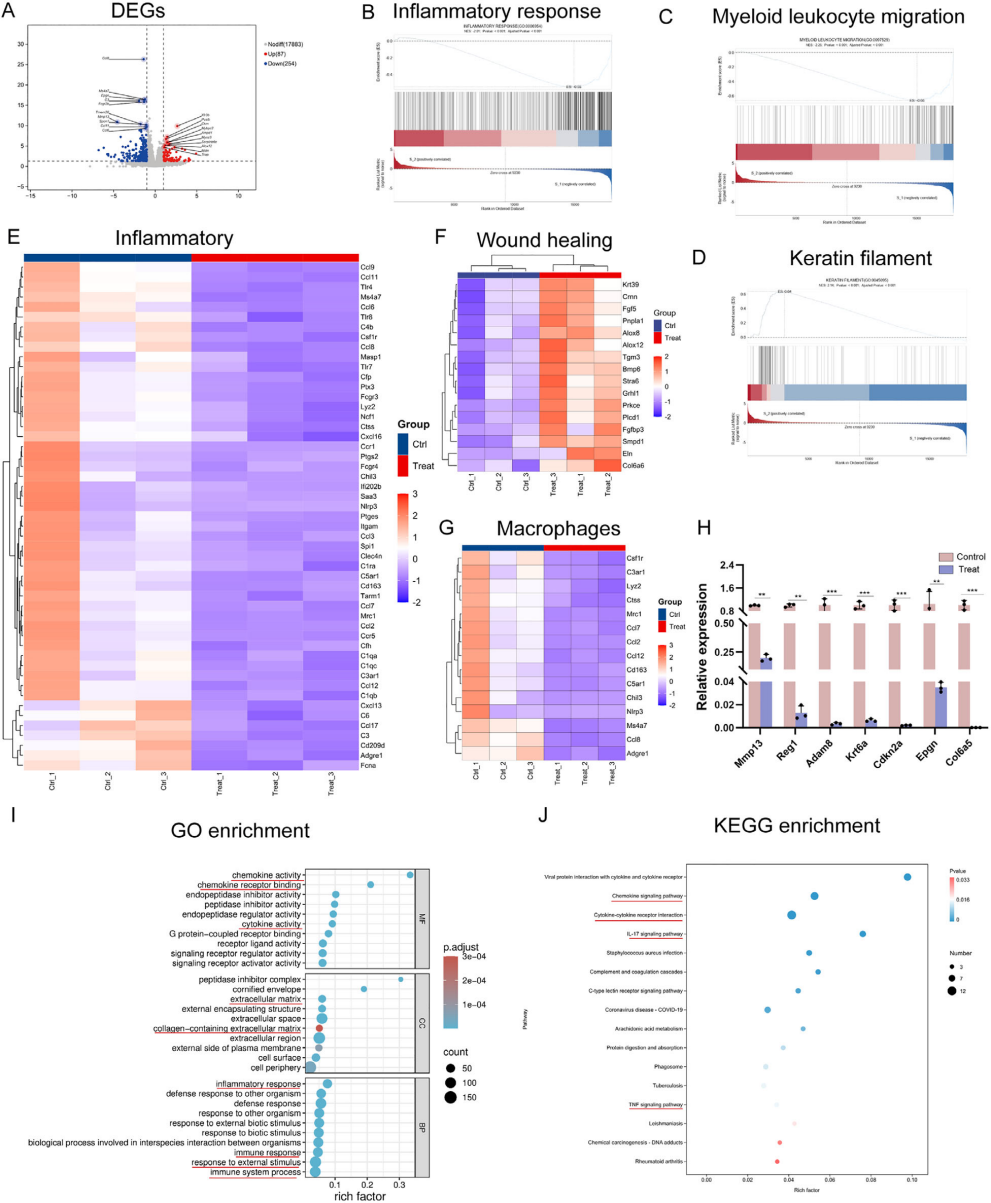
Day‐14 transcriptomic profiling reveals attenuation of inflammatory–myeloid programs and enrichment of epithelial and matrix‐remodeling signatures following Bil‐Gel@miR‐sEVs treatment. (A) Volcano plot of differentially expressed genes between infected control and Bil‐Gel@miR‐sEVs‐treated wound tissues. Differential expression was analyzed using DESeq2, with |log2 fold change| ≥ 1 and *p* < 0.05 as the screening criteria. (B–D) Gene set enrichment analysis (GSEA) of inflammatory response, myeloid leukocyte migration, and keratin filament gene sets. Normalized enrichment scores (NES) and nominal and adjusted p values are shown in the corresponding panels. (E–G) Heatmaps of representative inflammatory‐, repair‐, and macrophage/myeloid‐associated genes. Expression values are presented as row‐scaled Z‐scores. (H) qRT‐PCR validation of selected differentially expressed genes. Relative expression values are shown for visualization; statistical comparisons were performed on ΔCt values using two‐sided unpaired Student's t‐tests. (I, J) Gene Ontology (GO) and KEGG pathway over‐representation analyses, respectively; adjusted p‐values are represented by the color scale shown in the corresponding panels. RNA‐seq and qRT‐PCR analyses were performed using n = 3 independent biological samples per group. Quantitative validation data are presented as mean ± SD. ^*^ *p* < 0.05, ^**^ *p* < 0.01, ^***^ *p* < 0.001.

GSEA revealed a pronounced reduction in inflammatory‐ and myeloid‐associated transcriptional programs. In Bil‐Gel@miR‐sEVs‐treated wounds, the inflammatory response and myeloid leukocyte migration gene sets were negatively enriched, with normalized enrichment scores of −2.01 and −2.25, respectively, and adjusted *p*‐values < 0.001 (Figure 10B,C). In contrast, positive enrichment was observed for the keratin filament gene set(normalized enrichment score [NES] = 2.14, adjusted *p* < 0.001; Figure 10D). Together, these patterns were consistent with a day‐14 transcriptional state showing reduced inflammatory–myeloid activity alongside greater representation of epithelial structural programs. The stronger keratin‐associated signature also paralleled the improved epidermal organization observed histologically.

Heatmap analysis additionally showed coordinated downregulation of genes involved in innate immune activation, inflammatory mediator production, chemokine signaling, and myeloid responses, including members of the *Tlr*, *Ccl*, and *Fcgr* families as well as *Nlrp3*, *Ptgs2*, *Lyz2*, and *Ncf1* (Figure 10E,G). At the same time, transcripts associated with keratinization, cornification, epithelial differentiation, growth‐factor signaling, collagen organization, and extracellular‐matrix remodeling were increased in Bil‐Gel@miR‐sEVs‐treated wounds (Figure 10F). These gene‐level patterns further supported a transition from persistent inflammatory activity toward epithelial restoration and matrix remodeling.

GO analysis identified biological processes associated with cytokine and chemokine activity, inflammatory and immune responses, epithelial development, and extracellular‐matrix organization (Figure 10I). KEGG analysis further identified pathways involving chemokine signaling, cytokine–cytokine receptor interaction, IL‐17 and tumor necrosis factor (TNF) signaling, complement and coagulation cascades, phagosome‐related processes, and infection‐associated responses (Figure 10J). Because over‐representation analysis identifies pathway associations without establishing the direction of pathway activity, we interpreted these findings alongside the GSEA results and gene‐level expression patterns. qRT‐PCR independently reproduced the expression direction of selected inflammation‐ and repair‐associated genes identified by RNA sequencing (Figure 10H), validating representative transcriptional changes.

Because sequencing was conducted on whole‐wound tissue, the resulting RNA profiles reflect both changes in cellular composition and transcriptional regulation within individual cell populations. These data therefore cannot resolve cell‐type‐specific responses on their own. Nevertheless, the convergence of reduced inflammatory–myeloid signatures, increased epithelial and matrix‐remodeling transcripts, and corresponding histological changes supports a broader improvement in the wound tissue environment following Bil‐Gel@miR‐sEVs treatment.

Persistent inflammatory and myeloid activity characterizes impaired diabetic wound healing, whereas effective tissue repair requires restoration of epithelial organization, vascular remodeling, and extracellular‐matrix reconstruction [52, 53, 54, 55]. Here, the day‐14 transcriptome provided a molecular view of the later‐stage wound environment. Negative enrichment of inflammatory–myeloid programs was consistent with the earlier reductions in IRAK1, p‐p65, and IL‐6 and with the changes in macrophage‐associated tissue markers detected on day 7. Conversely, enrichment of keratin‐associated and matrix‐remodeling programs paralleled the improved epidermal organization, collagen deposition, vascularization, and stromal remodeling observed at the later stage. Thus, the transcriptomic findings complement the histological and molecular evidence for a transition toward tissue reconstruction.

Together with the preceding in vivo findings, the day‐14 transcriptomic profile provides molecular support for the stage‐adaptive therapeutic pattern observed throughout the study. Early photodynamic bacterial control was followed by attenuation of inflammatory signaling and changes in macrophage‐associated tissue markers, while the later wound state exhibited reduced inflammatory–myeloid activity together with enrichment of epithelial and matrix‐remodeling programs. Although these temporally separated measurements do not demonstrate a direct causal sequence, their concordance supports the stage‐adaptive design of Bil‐Gel@miR‐sEVs and its association with distinct biological responses across successive phases of infected diabetic wound healing.

### 2.11 Cytocompatibility, Hemocompatibility, and Preliminary Short‐Term In Vivo Safety

Using an indirect Transwell coculture system, we assessed the cytocompatibility of the blank hydrogel formulations. L929 fibroblasts and HUVECs exposed to Low‐Gel, Up‐Gel, or Bil‐Gel maintained high viability throughout the 3‐day observation period and showed no evident adverse morphological changes (Figure S8A,B,D–G). Across all hydrogel groups, cytoskeletal staining further showed well‐spread cell morphology with preserved filamentous actin organization (Figure S8H–K), supporting the indirect‐contact cytocompatibility of the constituent hydrogel networks.

We evaluated hemocompatibility using a red blood cell hemolysis assay. Relative to the positive control, Low‐Gel, Up‐Gel, and Bil‐Gel produced only minimal hemolysis, indicating favorable erythrocyte compatibility under the tested conditions (Figure S8C).

We further assessed short‐term in vivo tissue compatibility in a separate 10‐day subcutaneous implantation study using male C57BL/6J mice aged 8–10 weeks and weighing 25–27 g. Histological examination of the heart, liver, spleen, lungs, and kidneys showed no obvious material‐associated pathological abnormalities relative to the control group (Figure S8L).

Together, these findings support the cytocompatibility and hemocompatibility of the hydrogel formulations and provide preliminary evidence of short‐term in vivo safety.

## 3 Conclusion

We developed a stage‐adaptive bilayer hydrogel in which early photodynamic infection control is spatially separated from subsequent engineered‐sEV‐mediated immunoregulation and tissue repair. Rapid antibacterial and antibiofilm activity was provided by the Ce6‐containing wound‐contacting layer, whereas the ROS‐responsive upper compartment moderated excessive oxidative stress while serving as a reservoir for miR‐146a‐enriched engineered sEVs. During the early photodynamic phase, the bilayer architecture also restricted premature vesicle release. Following delivery, these engineered sEVs attenuated IRAK1/NF‐κB signaling, shifted macrophage‐associated marker profiles toward a more repair‐associated state, and retained reparative activity in endothelial cells and fibroblasts after incorporation into the hydrogel.

In infected diabetic wounds, Bil‐Gel@miR‐sEVs reduced bacterial burden while attenuating oxidative and inflammatory signals. At later stages, treatment was associated with improved epithelial reconstruction, collagen organization, vascularization, and stromal remodeling. Day‐14 transcriptomic profiling further revealed reduced inflammatory–myeloid programs alongside enrichment of epithelial and extracellular‐matrix remodeling signatures, consistent with a later‐stage wound environment more favorable for tissue reconstruction.

The defining feature of this platform is the spatiotemporal separation of distinct therapeutic functions rather than their concurrent delivery from a single compartment. By pairing an early photodynamic antibacterial phase with subsequent engineered‐sEV‐mediated immunoregulatory and reparative activity, the bilayer architecture aligns material function with the changing biological requirements of infected diabetic wound healing. Future translational development will require evaluation against a broader panel of multidrug‐resistant clinical isolates together with scalable and standardized sEV manufacturing.

## 4 Experimental Section

### 4.1 Materials and Reagents

Hyaluronic acid (HA; molecular weight, 800 kDa–1.0 MDa), poly(vinyl alcohol) 1799 (PVA 1799; degree of hydrolysis, 98–99 mol%), 1‐ethyl‐3‐(3‐dimethylaminopropyl)carbodiimide hydrochloride (EDC·HCl), N‐hydroxysuccinimide (NHS), poly(ethylene glycol) dipropiolate (PEG‐DA), thioketal‐containing linker (TK‐Linker), eight‐arm poly(ethylene glycol) thiol (8‐arm PEG‐thiol), and chlorin e6 (Ce6) were obtained from Aladdin Biochemical Technology Co., Ltd. (Shanghai, China). Sigma–Aldrich (St. Louis, MO, USA) supplied 3‐aminophenylboronic acid (3‐APBA).

Dulbecco's modified Eagle medium (DMEM), RPMI‐1640 medium, and fetal bovine serum (FBS) were supplied by Gibco (Life Technologies, USA). From Beyotime Biotechnology (Shanghai, China), we obtained radioimmunoprecipitation assay (RIPA) lysis buffer, the bicinchoninic acid (BCA) protein assay kit, DiO, crystal violet staining solution, the BeyoClick EdU assay kit, Hoechst 33342, phalloidin, and Calcein AM.

Proteintech Group, Inc. (Wuhan, China) supplied antibodies against CD9, CD81, TSG101, Calnexin, IRAK1, NF‐κB p65, phosphorylated NF‐κB p65 (p‐p65), and CD64. phycoerythrin (PE)‐conjugated anti‐human CD86 antibody (clone IT2.2, Cat. No. 305405) and allophycocyanin (APC)‐conjugated anti‐human CD206 antibody (clone 15‐2, Cat. No. 321110) were supplied by BioLegend (San Diego, CA, USA).

Dihydroethidium (DHE), antibodies for tissue immunostaining, and histological‐staining reagents were obtained from Servicebio Technology Co., Ltd. (Wuhan, China). Chemiluminescent horseradish peroxidase (HRP) substrate was obtained from Millipore, whereas the hydrogen peroxide assay kit was obtained from Solarbio. For reverse transcription and qPCR, we used HiScript III RT SuperMix for qPCR (+gDNA wiper; R323‐01) and SupRealQ Purple Universal SYBR qPCR Master Mix (U+; Q412‐02), respectively; both were obtained from Vazyme Biotech Co., Ltd. (Nanjing, China). Transparent film dressings were supplied by 3M.

### 4.2 Identification of miR‐146a and Generation and Characterization of Engineered sEVs

Human umbilical cord‐derived mesenchymal stromal cells (hUC‐MSCs; Cat. No. HUXUC‐01001) were obtained from Cyagen Biosciences Inc. At passages 3–5, cells were cultured at 37°C in a humidified atmosphere containing 5% CO_2_ using DMEM supplemented with EV‐depleted FBS; both media components were obtained from Gibco (Life Technologies, USA).

For candidate miRNA screening, hUC‐MSCs were cultured for 48 h in the presence or absence of 100 ng mL−^1^ lipopolysaccharide (LPS). After collection, conditioned medium was centrifuged at 10 000 × g for 30 min at 4°C, passed through a 0.22 µm membrane, and then ultracentrifuged at 100 000 × g for 3 h at 4°C using an Optima L‐100XP ultracentrifuge (Beckman Coulter, USA). The resulting vesicle pellets were resuspended in sterile PBS. sEVs obtained from untreated and LPS‐preconditioned hUC‐MSCs were designated MSC‐sEVs and LPS‐MSC‐sEVs, respectively. Independently prepared sEV samples were subjected to small RNA sequencing, followed by differential‐expression analysis and qRT‐PCR validation as detailed in the Supporting Information. Based on these analyses, miR‐146a‐5p was selected for subsequent engineering.

To generate engineered sEVs, we used the Sp‐scFv(hCD64)‐Gluc‐C1C2‐MS2 and PAC‐pri‐hsa‐miR‐146a‐PAC constructs. The Sp‐scFv(hCD64)‐Gluc‐C1C2‐MS2 construct included Gaussia luciferase (Gluc) as a reporter. Sequences S1 and S2 in the Supporting Information provide, respectively, the verified nucleotide sequence of the Sp‐scFv(hCD64)‐Gluc‐C1C2‐MS2 coding insert and the Sanger‐confirmed sequence region of the PAC‐pri‐hsa‐miR‐146a‐PAC construct.

The two constructs were packaged into recombinant lentiviral particles in HEK293T cells and used for co‐transduction of hUC‐MSCs. Transduction efficiency at MOIs of 10, 50, and 100 was assessed by GFP fluorescence imaging followed by quantification of the GFP‐positive area. Based on these measurements, an MOI of 50 was selected for subsequent engineered‐sEVs production. At 17 h after co‐transduction, fresh EVs‐depleted medium replaced the virus‐containing medium. Conditioned medium was then collected, and engineered sEVs were isolated using the centrifugation, filtration, and ultracentrifugation procedure described above. These vesicles were designated miR‐146a‐hCD64‐sEVs (miR‐sEVs). For the corresponding comparison group in subsequent experiments, sEVs were isolated from non‐engineered hUC‐MSCs and designated sEVs.

TEM, NTA, and Western blotting were used to characterize sEVs and miR‐sEVs. NTA was used to determine particle‐size distributions and concentrations, whereas vesicle protein concentrations were measured with the BCA assay. CD9, CD81, and TSG101 were assessed as EVs‐associated proteins, while Calnexin was examined as a non‐EV‐associated cellular protein. As a cellular control, hUC‐MSC lysate was included.

To evaluate particle yield, we used three independently prepared sEVs batches and three independently prepared miR‐sEVs batches (n = 3 per group). Producer‐cell numbers were recorded when conditioned medium was collected. Total particle recovery was calculated from the NTA‐determined particle concentration and final resuspension volume. Particle yield was then normalized to the corresponding producer‐cell number and expressed as particles per 10^6^ cells, with each independently prepared vesicle batch treated as one production replicate. We calculated the particle‐to‐protein ratio from the NTA‐determined particle concentration and corresponding BCA‐determined total protein concentration and expressed it as particles per µg of vesicle protein.

To assess reproducibility of miR‐146a enrichment, we analyzed three independently prepared miR‐sEVs batches. Total RNA was extracted separately from each preparation, and relative miR‐146a abundance was measured by qRT‐PCR using the reverse‐transcription and amplification procedures described in the Supporting Information. Each independently prepared miR‐sEVs batch represented one experimental replicate. Because miR‐146a is enriched by the MS2/pac system during vesicle biogenesis rather than through post‐isolation loading, miR‐146a content was reported as relative cargo enrichment across independent preparations rather than conventional encapsulation efficiency.

For cellular uptake analysis, sEVs and miR‐sEVs were incubated overnight with THP‐1 cells at 37°C. Following incubation, cells were washed with PBS, fixed in 4% paraformaldehyde for 15 min, and permeabilized with 0.2% Triton X‐100 for 10 min. Phalloidin and DAPI were then used to stain the cytoskeleton and nuclei, respectively, before cellular uptake was examined by confocal laser scanning microscopy.

To measure Gluc‐associated reporter activity, we used miR‐sEVs preparations at protein concentrations ranging from 0.1 to 100 µg mL−^1^. Bioluminescence was recorded under identical acquisition conditions, and linear regression was used to assess the relationship between miR‐sEVs protein concentration and Gluc‐associated signal intensity.

### 4.3 Fabrication and Physicochemical Characterization

HA‐PBA was synthesized through EDC/NHS‐mediated amidation. HA (1.0 g) was dissolved in 100 mL of deionized water, after which EDC·HCl (575 mg, 3.0 mmol) and NHS (345 mg, 3.0 mmol) were added while maintaining the pH at 5.0–6.0. Following activation for 20 min, 3‐APBA (410.65 mg, 3.0 mmol) was added, and the pH was readjusted to 5.0–6.0 using 1 M HCl. The reaction was then allowed to proceed overnight at room temperature. The resulting product was dialyzed against deionized water for 3 days using a membrane with a molecular‐weight cutoff of 3.5 kDa and subsequently lyophilized to obtain HA‐PBA.

To prepare Low‐Gel, HA‐PBA and PVA 1799 were separately dissolved in deionized water at concentrations of 2 and 9 wt.%, respectively. Ce6 was added to the HA‐PBA solution to achieve a final concentration of 10 µg mL−^1^ in Low‐Gel. The Ce6‐containing HA‐PBA solution was then combined with the PVA solution at a volume ratio of 2:1 and immediately vortexed to generate the dynamic boronate‐ester network.

For Up‐Gel preparation, poly(ethylene glycol) dipropiolate (PEG‐DA; 10 mg, 1 equivalent) and TK‐Linker (0.24 mg, 0.5 equivalents) were dissolved in 150 µL of PBS (pH 8.0) and mixed for 30 min at room temperature. Eight‐arm PEG‐thiol (6.25 mg, 0.125 equivalents), separately dissolved in 150 µL of PBS, was subsequently added to form the upper hydrogel network. For vesicle‐loaded formulations, sEVs or miR‐sEVs were incorporated into the Up‐Gel precursor before gelation to a final vesicle‐protein concentration of 100 µg mL−^1^. To ensure dose consistency among comparison groups, the same vesicle‐protein concentration was maintained in all sEV‐containing formulations. The resulting preparations were designated Up‐Gel@sEVs and Up‐Gel@miR‐sEVs, respectively.

To assemble the bilayer hydrogel, the Up‐Gel precursor was applied directly to the surface of preformed Low‐Gel and allowed to gel in situ. A 1:1 volume ratio of Up‐Gel to Low‐Gel was maintained. The assembled bilayer hydrogels were then subjected to three freeze–thaw cycles, with each cycle comprising freezing at −20°C for 30 min followed by thawing at 4°C for 2 h. The blank bilayer hydrogel was designated Bil‐Gel, whereas formulations containing sEVs or miR‐sEVs in the upper compartment were designated Bil‐Gel@sEVs and Bil‐Gel@miR‐sEVs, respectively.

### 4.4 Physicochemical and Mechanical Characterization of the Bilayer Hydrogel and Its Components

The internal morphologies of Low‐Gel and Up‐Gel and the Low‐Gel/Up‐Gel interface within Bil‐Gel were examined by scanning electron microscopy (SEM; SU8010, Hitachi, Japan). Freeze‐dried samples were sectioned to expose their internal structures. Bil‐Gel was sectioned perpendicular to the interface, allowing both hydrogel compartments to be visualized within a single section. Specimens were mounted on conductive carbon tape, sputter‐coated with gold under vacuum, and imaged at the indicated magnifications. Apparent pore sizes were quantified from SEM images using ImageJ. Because these measurements were performed on freeze‐dried specimens, the resulting values represent apparent pore dimensions rather than hydrated network mesh sizes.

Chemical characterization was carried out using proton nuclear magnetic resonance (^1^H NMR) spectroscopy and Fourier‐transform infrared (FTIR) spectroscopy. HA‐PBA was dissolved in D_2_O and analyzed at 296.9 K using a 400.13 MHz NMR spectrometer (Bruker BioSpin, Germany). Spectra were acquired using 32 transients and processed with TopSpin 3.5 pl 7 software. TK‐Linker, poly(ethylene glycol) dipropiolate (PEG‐DA), eight‐arm PEG‐thiol, and the corresponding upper‐layer formulation were likewise characterized by ^1^H NMR. FTIR spectra were acquired over 4000–600 cm−^1^ at a resolution of 4 cm−^1^ using 32 scans per measurement.

Rheological measurements were conducted at 25°C using a Discovery HR‐2 rotational rheometer (TA Instruments, USA) equipped with a 20 mm parallel‐plate geometry. The linear viscoelastic region was first established by amplitude sweeps, after which frequency sweeps were performed from 0.1 to 100 rad s−^1^ within this region. Steady‐shear measurements were subsequently conducted over a shear‐rate range of 0.1–100 s−^1^.

The macroscopic structural recovery of Low‐Gel was assessed qualitatively using a cut‐and‐contact test. Freshly prepared Low‐Gel was completely divided into two pieces using a sterile scalpel, and the newly exposed surfaces were immediately repositioned in contact without additional adhesive or external crosslinking. Recovery of visible structural continuity was documented photographically at the indicated time points.

For swelling measurements, freshly prepared Low‐Gel, Up‐Gel, and Bil‐Gel samples were weighed to obtain the initial wet mass (*W_0_*). To do so, these were individually immersed in 10 mL of PBS at 37°C. Next, at predefined time points up to 48 h, samples were removed and gently blotted with filter paper to eliminate excess surface liquid and avoid compression. Samples were then immediately weighed (*W_t_*) before being returned to the corresponding PBS. The relative swelling ratio was calculated as:

$$Relative\;swelling\;ratio\left(\%\right)=\frac{W_{t}-W_{0}}{W_{0}}\times 100\%$$

Here, *W*_0_ and *W_t_* represent the initial wet mass and the wet mass at time t, respectively. Five independently prepared samples were analyzed for each formulation (n = 5).

Interfacial adhesion between the Low‐Gel and Up‐Gel compartments was evaluated using a universal mechanical testing system (Instron 5967, Instron, USA). Bil‐Gel specimens with a defined Low‐Gel/Up‐Gel interfacial area of 1 cm^2^ were tested either immediately after preparation or following incubation in PBS at 37°C for 24 h. The free ends of both hydrogel layers were fixed to rigid supporting tabs and tested in a lap‐shear configuration at a crosshead speed of 5 mm min−^1^. Loading continued until either interfacial separation or bulk hydrogel failure occurred. From the resulting force–displacement curve, the maximum force (F_max_) was determined, and interfacial adhesion strength was calculated as:

$$\sigma_{\mathrm{interface}}=\frac{F_{\max}}{A}$$

Here, F_max_ denotes the maximum force, whereas A denotes the initial interfacial area. Following testing, failure modes were classified as adhesive, cohesive, or mixed. For each condition, three independently prepared specimens were analyzed (n = 3).

Wet‐tissue adhesion was quantified using freshly excised porcine skin under a lap‐shear configuration. Before testing, the tissue was maintained in a moist state. Low‐Gel or Bil‐Gel was brought into contact with the tissue over a defined area of 1 cm^2^ and subjected to a 200 g preload for 5 min to standardize hydrogel–tissue contact. The specimens were subsequently mounted on an Instron 5967 universal mechanical testing system and loaded in shear at a crosshead speed of 5 mm min−^1^ until either interfacial separation or hydrogel failure occurred. The maximum force (F_max_) was determined from the force–displacement curve, and wet‐tissue adhesion strength was calculated as:

$$\sigma_{\mathrm{tissue}}=\frac{F_{\max}}{A}$$

Here, A represents the initial hydrogel–tissue contact area. Five independently prepared specimens were analyzed for each formulation (n = 5).

Hydrogel degradation under oxidative conditions was evaluated by monitoring the remaining wet mass. Low‐Gel samples with an initial wet mass of 2 g were immersed at 37°C in 10 mL of PBS alone or PBS containing 50, 100, 500, or 1000 µM H_2_O_2_ and assessed at 0, 24, 48, 72, 96, and 120 h. Up‐Gel samples with the same initial wet mass were incubated at 37°C in 10 mL of PBS alone or PBS containing 100 or 500 µM H_2_O_2_ and assessed at 0, 12, 24, 48, 72, 120, 168, 240, and 336 h. At each time point, samples were retrieved and gently rinsed with deionized water to remove residual salts. Excess surface liquid was then removed by gentle blotting with filter paper without compressing the hydrogel, and the samples were immediately weighed. The remaining wet mass was calculated as:

$$\mathrm{Remaining}\;\mathrm{wet}\;\mathrm{mass}\;\left(\%\right)=\frac{W_{t}}{W_{0}}\times 100\%$$

Here, *W_0_* represents the initial wet mass, and *W_t_* represents the wet mass at time *t*. We analyzed three independently prepared samples for each condition (n = 3).

### 4.5 Release of Engineered sEV‐Associated Particles and Characterization of Released Vesicle‐Associated Fractions

The release of engineered sEV‐associated particles from the hydrogel formulations was assessed using a Transwell‐based system. Up‐Gel@miR‐sEVs and Bil‐Gel@miR‐sEVs were prepared as described above. Bil‐Gel@miR‐sEVs was oriented such that the Low‐Gel compartment faced the porous membrane and receiver compartment, with the miR‐sEV‐containing Up‐Gel compartment positioned above it. Samples were placed in 6‐well Transwell inserts fitted with 0.4 µm porous membranes, and 2.6 mL of sterile PBS (pH 7.4) was added to the receiver compartment before incubation at 37°C. For the PDT‐treated condition, Bil‐Gel@miR‐sEVs was exposed to 660 nm light at a power density of 25 mW cm−^2^ for 10 min. Nonirradiated samples were subjected to identical handling without light exposure.

Release fractions were collected at 12 h and on days 1, 3, 7, and 14. At each sampling point, 200 µL of receiver medium was removed and immediately replaced with an equal volume of fresh PBS. Particle concentrations in the collected fractions were measured by NTA. Cumulative particle release was corrected for particles removed during serial sampling and calculated as:

$$Q_{n}=\frac{C_{n}V+\sum_{i=1}^{n-1}C_{i}V_{s}}{N_{0}}\times 100\%$$

Here, *Qn* represents the cumulative percentage of NTA‐detectable particles released at sampling point n, *Cn* is the NTA‐determined particle concentration at any sampling point *n*, *V* is the total receiver‐medium volume (2.6 mL), *Ci* is the particle concentration measured at each preceding sampling point, *Vs* is the sampling volume (0.2 mL), and *N_0_* represents the initial number of NTA‐detectable particles that was incorporated into each hydrogel sample. Interval particle release was calculated by computing the difference between consecutive cumulative release values.

To assess vesicle‐associated miR‐146a after release, fractions collected on days 1 and 14 were first normalized to equal numbers of NTA‐detectable particles before RNA extraction. The original miR‐sEV preparation was processed in parallel and served as the reference. Total RNA was then extracted from the normalized samples, and relative miR‐146a abundance was quantified by qRT‐PCR using the reverse‐transcription and amplification procedures detailed in the Supporting Information.

For Western blot analysis, day‐1 and day‐14 release fractions and the original miR‐sEV preparation were normalized to equal amounts of total protein before electrophoresis. CD9, CD81, and TSG101 were analyzed as EV‐associated proteins, whereas Calnexin was evaluated as a non‐EV‐associated cellular protein. Where indicated, band intensities were quantified using ImageJ.

For morphological characterization, vesicle‐associated particles recovered from the day‐7 release fractions were concentrated as needed and subsequently examined by TEM using the sample‐preparation and imaging procedures described above. For each condition, three independently prepared samples were analyzed (n = 3).

### 4.6 Ce6 Release, Redox‐Regulatory Properties, and Cytoprotective Effects

Ce6 release from the hydrogel formulations was assessed under oxidative conditions. Low‐Gel and Bil‐Gel samples were immersed in 10 mL of PBS containing 1000 µM H_2_O_2_ and incubated at 37°C with orbital shaking at 400 rpm. At 2, 4, 6, 8, 10, and 12 h, 200 µL of release medium was withdrawn and immediately replaced with an equal volume of fresh medium, with the 0 h measurement serving as the baseline. Ce6 concentrations in the collected fractions were determined from absorbance at 660 nm using an established calibration curve. Cumulative release was corrected for material removed during serial sampling and expressed as a percentage of the initial Ce6 loading.

Photoinduced oxidative activity was initially assessed using a DCFH‐based assay. Hydrogel‐derived fractions were incubated with the probe and then exposed to 660 nm light for the indicated durations. DCF‐associated fluorescence was measured under identical acquisition settings.

Photoinduced oxidative activity was additionally evaluated using a TMB oxidation assay. Low‐Gel, Up‐Gel, and Bil‐Gel samples (50 mg each) were separately added to reaction mixtures containing TMB at a final concentration of 4 mM, while a TMB‐containing reaction mixture without hydrogel served as the control. Samples were exposed to 660 nm light at a power density of 25 mW cm−^2^ for 10 min and then incubated at 37°C for 30 min. Except during irradiation, reaction mixtures were protected from strong ambient light. UV–visible absorption spectra were acquired over 400–800 nm, and the characteristic absorbance of oxidized TMB at approximately 652 nm was compared across groups. The DCFH and TMB assays served as nonspecific readouts of overall photoinduced oxidative activity rather than as methods for identifying or quantifying individual ROS species.

Radical‐scavenging properties were assessed using DPPH and ABTS assays. For the DPPH assay, hydrogel samples (50 mg) were immersed in 5 mL of ethanol and incubated in the dark for 4 h. A 500 µL aliquot of the resulting supernatant was combined with 500 µL of 0.1 mM DPPH solution in ethanol and incubated in the dark for 30 min. Subsequently, 200 µL of each reaction mixture was transferred to a 96‐well plate, and absorbance at 517 nm was measured using a Synergy H1 multimode microplate reader (BioTek, Winooski, VT, USA). DPPH radical‐scavenging activity was calculated as:

$$DPPH\;scavenging\;activity\left(\%\right)=\frac{A_{control}-A_{sample}}{A_{control}}\;\times 100\%$$

Here the terms A_control_ and A_sample_ represent the absorbance values of the control and sample‐containing reactions, respectively.

For the ABTS assay, hydrogel samples (50 mg) were immersed in 5 mL of ethanol and incubated in the dark for 4 h. To generate the ABTS radical working solution, 7 mM ABTS solution was combined with 2.4 mM potassium persulfate at a 1:1 volume ratio and incubated at 4°C in the dark for 12 h. The hydrogel extracts were subsequently mixed with the ABTS working solution at a 1:5 volume ratio and incubated at 37°C in the dark for 30 min. Absorbance at 734 nm was then measured using a UV‐2600 UV–visible spectrophotometer (Shimadzu, Japan). ABTS radical‐scavenging activity was calculated as:

$$ABTS\;scavenging\;activity\left(\%\right)=\frac{A_{control}-A_{sample}}{A_{control}}\times 100\%$$

Here, A_control_ and A_sample_ represent the absorbance values of the control and sample‐containing reactions, respectively.

The effect of hydrogel treatment on H_2_O_2_‐associated oxidative activity was assessed using a TMB‐based colorimetric assay. Low‐Gel, Up‐Gel, and Bil‐Gel samples (50 mg each) were separately incubated at 37°C for 2 h with 1 mL of 2 mM H_2_O_2_. H_2_O_2_ incubated under identical conditions without hydrogel served as the control. After the hydrogels were removed, TMB was added to the collected supernatants to a final concentration of 1 mM, followed by incubation at 37°C for 10 min while protected from strong ambient light. UV–visible absorption spectra were then acquired over 400–800 nm, and formulation‐dependent differences in the characteristic TMB absorbance at approximately 652 nm were compared among groups. This assay served as a comparative readout of H_2_O_2_‐associated oxidative activity rather than a direct quantitative measurement of residual H_2_O_2_.

For cellular oxidative‐stress analysis, L929 fibroblasts, HUVECs, and PMA‐differentiated THP‐1 macrophages were challenged with 200 µM H_2_O_2_ for 3 h while exposed to the indicated hydrogel formulations using an indirect‐contact configuration. Intracellular redox‐sensitive fluorescence was then assessed using DCFH‐DA according to the manufacturer's instructions. Within each cell type, images were acquired under identical settings, and fluorescence intensity was quantified following background subtraction.

Cytoprotective effects were further assessed in H_2_O_2_‐challenged L929 fibroblasts. Cells were stained with Calcein AM and PI and subsequently imaged using an IX83 inverted fluorescence microscope (Olympus, Japan). In parallel, mitochondrial membrane potential was evaluated using JC‐1 staining. Red and green fluorescence images were acquired under identical settings, after which the ratio of red JC‐1 aggregate fluorescence to green JC‐1 monomer fluorescence was calculated. Unless otherwise indicated, three independent experiments were conducted for each condition (n = 3), and technical measurements obtained within the same independent experiment were averaged before statistical analysis.

### 4.7 Photodynamic Antibacterial and Antibiofilm Activity In Vitro

Photodynamic antibacterial activity of the hydrogel formulations was evaluated against *Staphylococcus aureus* (ATCC 25923, American Type Culture Collection, Manassas, VA, USA), *Escherichia coli* (ATCC 25922, American Type Culture Collection, Manassas, VA, USA), and a multidrug‐resistant *Pseudomonas aeruginosa* strain (ATCC BAA‐2108, American Type Culture Collection, Manassas, VA, USA). The multidrug‐resistant phenotype of *P. aeruginosa* ATCC BAA‐2108 was documented by the strain provider. Sterilized hydrogel samples were incubated in 24‐well plates with bacterial suspensions at 1 × 10^6^ CFU mL−^1^. The designated groups were exposed to 660 nm light at a power density of 25 mW cm−^2^ for 10 min, whereas matched nonirradiated groups were handled identically without light exposure. Following incubation at 37°C for 24 h, bacterial suspensions were serially diluted, and 100 µL of the appropriate dilution was plated onto Luria–Bertani agar. After an additional 24 h of incubation at 37°C, colonies were counted. Relative bacterial viability was calculated by normalizing CFU counts to the corresponding untreated control.

The effects of the hydrogel formulations on biofilm formation and pre‐established biofilms were assessed by crystal violet staining. Suspensions of *S. aureus*, *E. coli*, or *P. aeruginosa* at 1 × 10^7^ CFU mL−^1^ were seeded into 96‐well plates at a final volume of 200 µL per well. For the biofilm formation assay, hydrogel formulations were introduced at the beginning of bacterial incubation. For the pre‐established biofilm assay, bacteria were first cultured for 48 h to allow biofilm formation. The planktonic phase was subsequently removed carefully, and the adherent biofilms were gently washed with PBS before the hydrogel formulations were added.

For both assays, designated groups were exposed to 660 nm light at a power density of 25 mW cm−^2^ for 10 min and then incubated at 37°C with orbital shaking at 120 rpm for 36 h. Planktonic bacteria were removed, after which the wells were washed three times with PBS and stained with 0.1% crystal violet for 10 min. Excess stain was removed, and the plates were allowed to air‐dry. Retained crystal violet was subsequently solubilized in 100 µL of 95% ethanol for 30 min under gentle orbital agitation. Absorbance at 570 nm was measured using a Synergy H1 multimode microplate reader (BioTek, Winooski, VT, USA). Biofilm formation was quantified relative to the corresponding untreated control. For the pre‐established biofilm assay, residual biofilm biomass was similarly normalized to the corresponding untreated control. Three independent experiments were conducted for each condition.

Biofilm‐associated bacterial viability and architecture were further evaluated using SYTO9/PI staining. After treatment, biofilms were gently washed with PBS and stained according to the manufacturer's instructions. Fluorescence images were acquired under identical settings across groups, and Z‐stack images were collected for three‐dimensional reconstruction. Changes in bacterial membrane integrity and biofilm organization were assessed from the relative distributions of SYTO9‐ and PI‐associated fluorescence.

Bacterial morphology following treatment was examined by field‐emission scanning electron microscopy. Bacterial suspensions at 1 × 10^7^ CFU mL−^1^ were incubated with the indicated hydrogel formulations under the corresponding irradiation conditions. Following incubation at 37°C for 12 h, bacteria were collected, washed with PBS, and fixed overnight in 2.5% glutaraldehyde at 4°C. Samples were then dehydrated through a graded ethanol series (30%–100%; 15 min at each concentration), deposited onto silicon wafers, critical‐point dried, and sputter‐coated with Au/Pd. Morphological changes were evaluated using a field‐emission scanning electron microscope (SU8010, Hitachi, Japan) at an accelerating voltage of 5 kV.

### 4.8 CD64 Blockade and IRAK1/NF‐κB Signaling Analysis in Macrophages

THP‐1 cells were maintained in RPMI‐1640 complete medium and differentiated into macrophage‐like cells by exposure to 100 ng mL−^1^ phorbol 12‐myristate 13‐acetate (PMA) for 24 h. The PMA‐containing medium was then replaced with fresh complete medium, followed by a further 24 h resting period. CD64 expression was assessed by flow cytometry before and after PMA‐induced differentiation.

Before subsequent treatment, differentiated macrophages were stimulated with 100 ng mL−^1^ lipopolysaccharide (LPS) for 24 h. Four experimental groups were examined: NC, untreated macrophages without LPS stimulation; Control, macrophages treated with LPS alone; miR‐sEVs, LPS‐stimulated macrophages subsequently treated with miR‐sEVs; and αCD64 + miR‐sEVs, LPS‐stimulated macrophages subjected to CD64 blockade before treatment with miR‐sEVs. For receptor blockade, cells were preincubated for 1 h with 10 µg mL−^1^ anti‐human CD64 antibody (clone 10.1) and then treated for 24 h with miR‐sEVs at a final vesicle‐protein concentration of 100 µg mL−^1^.

For analysis of macrophage surface markers, cells were stained according to the manufacturer's instructions with PE‐conjugated anti‐human CD86 antibody (clone IT2.2, BioLegend, Cat. No. 305405) and APC‐conjugated anti‐human CD206 antibody (clone 15‐2, BioLegend, Cat. No. 321110). Flow‐cytometric data were acquired using a FACSCanto II flow cytometer (BD Biosciences, USA) and analyzed using FlowJo v10.0.7r2 (Tree Star, Inc., Ashland, OR, USA). Cellular debris and aggregates were excluded based on forward‐ and side‐scatter characteristics together with singlet gating. The percentages of CD86‐positive and CD206‐positive cells were determined, after which the CD86‐positive/CD206‐positive cell ratio was calculated.

Intracellular miR‐146a abundance and mRNA expression levels of *IRAK1*, *TRAF6*, *RELA*, and *NFKBIA* were determined by qRT‐PCR according to the general procedure described above. miR‐146a abundance was normalized to U6, whereas mRNA expression levels were normalized to GAPDH. Relative expression was calculated using the 2−ΔΔCt method. Primer sequences are provided in Table S1.

IRAK1/NF‐κB signaling was additionally examined by Western blotting. Total cellular protein was extracted using RIPA lysis buffer supplemented with protease and phosphatase inhibitors, followed by quantification using the BCA assay. Equal amounts of protein (30 µg per lane) were separated bysodium dodecyl sulfate–polyacrylamide gel electrophoresis (SDS‐PAGE) and transferred by wet electrotransfer onto 0.22 µm polyvinylidene fluoride (PVDF) membranes. Membranes were blocked for 1 h with 5% bovine serum albumin (BSA) in Tris‐buffered saline containing Tween 20 (TBST) and then incubated overnight at 4°C with primary antibodies against IRAK1, phosphorylated NF‐κB p65 (p‐p65), total NF‐κB p65, and GAPDH. Following incubation with HRP‐conjugated secondary antibodies for 1 h at room temperature, protein bands were visualized by enhanced chemiluminescence and quantified using ImageJ. IRAK1 abundance was normalized to GAPDH, whereas NF‐κB activation was evaluated from the p‐p65/total p65 ratio. Full, uncropped membrane images are provided in Figure S9. Finally, for each group we used three independent cell cultures (n = 3) for each analysis.

### 4.9 Cytocompatibility, Hemocompatibility, and Preliminary Short‐Term In Vivo Safety Analyses

Next, the cytocompatibility of Low‐Gel, Up‐Gel, and Bil‐Gel was assessed using L929 fibroblasts and HUVECs in an indirect Transwell coculture system. Cellular metabolic activity was quantified using the Cell Counting Kit‐8 (CCK‐8) assay, whereas cell viability was evaluated by live/dead staining on days 1, 2, and 3. After 24 h of indirect coculture, cytoskeletal organization and cell spreading were examined by phalloidin staining.

Hemocompatibility was assessed using hydrogel extracts generated by incubating each formulation in 1 mL of saline at 37°C for 12 h. Fresh anticoagulated goat blood (20 µL) was then added to each extract and incubated at 37°C with orbital shaking at 120 rpm for 2 h. Following incubation, samples were centrifuged at 200 × g for 10 min, and hemoglobin release into the supernatant was quantified by measuring absorbance at 545 nm. Deionized water and saline containing the same volume of blood served as positive and negative controls, respectively.

For preliminary short‐term in vivo safety assessment, 0.5 g of Low‐Gel, Up‐Gel, or Bil‐Gel was surgically implanted into the subcutaneous tissue of male C57BL/6J mice aged 8–10 weeks and weighing 25–27 g. Each group included three mice (n = 3). At 10 days after implantation, the heart, liver, spleen, lungs, and kidneys were collected and processed for H&E staining.

### 4.10 In Vitro Endothelial and Fibroblast Repair‐Associated Responses

Experimental groups included Control, Low‐Gel, Up‐Gel, Free miR‐sEVs, Up‐Gel@miR‐sEVs, Bil‐Gel, and Bil‐Gel@miR‐sEVs. For indirect coculture assays, hydrogel formulations were placed in Transwell inserts while cells were cultured in the lower compartments; free miR‐sEVs were added directly to the culture medium. The nominal sEV protein dose used for the Free miR‐sEV group was matched to the dose loaded into Up‐Gel@miR‐sEVs.

Cell proliferation was evaluated using an EdU incorporation assay. HUVECs and L929 fibroblasts were seeded in the lower compartments of Transwell plates and exposed to the indicated treatments for 24 h. Cells were subsequently labeled with EdU, fixed, permeabilized, and stained according to the assay protocol. Fluorescence images were acquired using an IX83 inverted microscope (Olympus, Japan), and proliferation was quantified as the percentage of EdU‐positive nuclei relative to the total number of nuclei.

For scratch assays, HUVECs and L929 fibroblasts were seeded into sixwell plates at 1 × 10^6^ cells mL−^1^ and cultured for 24 h to establish confluent monolayers. A linear scratch was then generated using a sterile 200 µL pipette tip, after which detached cells were removed by gentle washing with PBS. The indicated treatments were subsequently applied, and the same scratched regions were imaged at 0, 12, and 24 h using the IX83 inverted microscope. Scratch areas were quantified using ImageJ v1.53, and scratch closure was calculated as:

$$Scratch\;closure\left(\%\right)=\frac{A_{0}-A_{t}}{A_{0}}\times 100\%$$

Here, A_0_ and A_t_ represent the scratch areas obtained 0 h and at time t, respectively.

Cell migration was additionally evaluated using Transwell chambers. HUVECs or L929 fibroblasts suspended in serum‐free medium were seeded into the upper chambers, whereas the corresponding treatments were applied to culture medium in the lower compartments. After 24 h, nonmigrated cells remaining on the upper membrane surface were gently removed. Cells that had migrated to the lower surface were then fixed with 4% paraformaldehyde, stained with crystal violet, imaged using the IX83 inverted microscope, and quantified with ImageJ.

Endothelial network formation was assessed using a Matrigel‐based tube‐formation assay. Growth‐factor‐reduced Matrigel (50 µL per well) was dispensed into prechilled 96‐well plates and allowed to polymerize for 1 h at 37°C in a humidified atmosphere containing 5% CO_2_. Following 24 h of the indicated treatments, HUVECs were collected and seeded onto the polymerized Matrigel at 4 × 10^4^ cells per well. After 3 h, phase‐contrast images were acquired at 10× magnification using the IX83 inverted microscope. Three non‐overlapping fields were analyzed from each well, and junction and mesh numbers were quantified using ImageJ v1.53 with the Angiogenesis Analyzer plugin.

VEGF expression in HUVECs and collagen I/III expression in L929 fibroblasts were evaluated by immunofluorescence. After 24 h of treatment, cells were fixed with 4% paraformaldehyde for 15 min, permeabilized with Triton X‐100, and then blocked for 1 h at room temperature. HUVECs were incubated overnight at 4°C with a primary antibody against VEGF, whereas L929 fibroblasts were incubated with primary antibodies against collagen I or collagen III. Following washing, cells were incubated with the corresponding fluorophore‐conjugated secondary antibodies for 1 h at room temperature in the dark and then counterstained with DAPI. Fluorescence images were obtained using the IX83 inverted microscope, and mean fluorescence intensity was quantified with Fiji/NIH ImageJ.

For all imaging‐based assays, acquisition settings, including objective magnification, exposure time, detector gain, and illumination intensity, were maintained consistently across treatment groups within the same experiment. At least three non‐overlapping fields were collected from each independent biological replicate using a predefined field‐selection procedure. Measurements obtained from multiple fields were averaged to yield one value for each biological replicate, and three independent biological replicates were analyzed in each group (n = 3).

### 4.11 Establishment and Treatment of Infected Diabetic Wounds

All animal procedures were approved by the Animal Ethics Committee of the Second Affiliated Hospital of Kunming Medical University (Approval No. kyfey2026055). Male C57BL/6J mice aged 8–10 weeks and weighing 25–27 g were obtained from Beijing SPF Biotechnology Co., Ltd. (Beijing, China) and housed under specific pathogen‐free conditions.

Following a 12 h fast, diabetes was induced by once‐daily intraperitoneal administration of freshly prepared streptozotocin (STZ; 50 mg kg−^1^) dissolved in 0.1 M citrate buffer (pH 4.5) for five consecutive days. Blood glucose was measured using tail‐vein blood, and mice exhibiting glucose concentrations ≥ 16.7 mmol L−^1^ on three consecutive measurements were classified as diabetic.

After diabetes was confirmed, mice were anesthetized with 2,2,2‐tribromoethanol (250 mg kg−^1^, intraperitoneally), and a single 8‐mm full‐thickness wound was created on the dorsal skin of each animal. Suspensions of *Staphylococcus aureus*, *Escherichia coli*, and the same MDR *P. aeruginosa* strain used in the in vitro antibacterial assays were combined in equal volumes (1:1:1, v/v/v) to produce a final total bacterial concentration of 1 × 10^8^ CFU mL−^1^. Each wound was inoculated with 10 µL of the mixed bacterial suspension, corresponding to 1 × 10^6^ CFU per wound, and infection was allowed to establish for 24 h before treatment. An 8‐mm‐diameter circular hydrogel dressing was subsequently applied to each wound. For sEV‐containing formulations, each dressing contained a nominal sEV protein loading of approximately 5 µg per wound.

A total of 96 mice were included. The primary therapeutic cohort consisted of 90 mice assigned to six groups (n = 15 per group): I, infected control; II, Low‐Gel; III, Up‐Gel@miR‐sEVs; IV, Bil‐Gel; V, Bil‐Gel@sEVs; and VI, Bil‐Gel@miR‐sEVs. A separate transcriptomic cohort comprised six additional mice assigned to the infected control and Bil‐Gel@miR‐sEVs groups (n = 3 per group). Animals in this transcriptomic cohort received the same corresponding treatments as those assigned to the primary therapeutic cohort.

Immediately after hydrogel application, groups II, IV, V, and VI were irradiated with 660 nm light at a power density of 100 mW cm−^2^ for 10 min, whereas groups I and III were not irradiated. The higher irradiance used in vivo relative to the in vitro experiments was selected to compensate for optical attenuation and scattering at the wound surface. Following treatment, wounds were covered with Tegaderm transparent film to protect the wound area and retain the applied formulations.

Wound progression in the primary therapeutic cohort was documented on days 0, 3, 7, 10, and 14. For longitudinal analysis of wound closure, the same three mice from each group were followed through day 14 and photographed at every time point. Wound areas were quantified using NIH ImageJ.

On day 3, nine mice from each group in the primary therapeutic cohort were euthanized for independent early‐stage assessments. Fresh wound tissues from three mice per group were collected aseptically, homogenized in sterile PBS, serially diluted, and plated for colony‐forming unit analysis to determine bacterial burden. Three different mice per group were used for dihydroethidium (DHE) fluorescence analysis, while another three mice per group were used for histological evaluation.

On day 7, three additional mice from each group were euthanized for intermediate‐stage tissue analyses. Wound tissue from each animal was divided between histological and immunofluorescence analyses and Western blotting. The remaining three mice in each group of the primary therapeutic cohort were followed through day 14 and used for terminal histological and immunofluorescence analyses.

On day 14, the six mice assigned to the independent transcriptomic cohort were euthanized. Wound tissues were then collected for bulk RNA sequencing and subsequent transcriptome‐related molecular validation. Each individual mouse was treated as one independent biological replicate.

### 4.12 Wound Closure and DHE Fluorescence Analyses

Digital photographs of wounds were acquired immediately before treatment (day 0) and on days 3, 7, 10, and 14 under standardized imaging conditions, with a scale ruler included in every image. Wound margins were delineated, after which wound areas were quantified using NIH ImageJ. At each time point, wounds from three mice per group were analyzed (n = 3 per time point). For each mouse, wound closure was calculated relative to its corresponding pretreatment wound area on day 0 as follows:

$$\mathrm{Wound}\;\mathrm{closure}\;(\%)=\left(A_{0}-A_{t}\right)/A_{0}\times 100\%$$

Here A_0_ represents the wound area immediately before treatment on day 0, whereas A_t_ represents the wound area at the indicated time point.

On day 3, DHE‐detectable oxidative activity in wound tissue was evaluated using dihydroethidium (DHE) staining. Cryosections were prepared and stained according to the manufacturer's instructions. Across all groups, fluorescence images were acquired using identical exposure and acquisition settings. Following background subtraction, integrated DHE‐associated fluorescence within predefined wound‐tissue regions of interest was quantified using ImageJ and normalized to the corresponding region‐of‐interest area to determine mean fluorescence intensity. Three independent biological replicates were analyzed for each group (n = 3).

### 4.13 Histological and Immunofluorescence Analyses of Wound Tissues

For histological analysis, wound tissues were fixed in 4% paraformaldehyde, embedded in paraffin, and sectioned at a thickness of 5 µm. Sections were stained with H&E and Masson's trichrome. Neoepidermal thickness and collagen‐positive area were subsequently quantified using ImageJ. Measurements obtained from multiple predefined positions or non‐overlapping fields within each wound were averaged to generate a single value for each animal.

For immunofluorescence analysis, wound sections collected on day 7 were stained for IRAK1, phosphorylated NF‐κB p65 (p‐p65), IL‐6, CD86, and CD163, whereas sections collected on day 14 were stained for CD31, α‐smooth muscle actin (α‐SMA), VEGF, and Ki67. CD86 and CD163 served as macrophage‐associated tissue markers, while CD31 and α‐SMA were used to evaluate neovascularization and vascular remodeling.

Within each staining experiment, identical imaging parameters were used to acquire fluorescence images across all treatment groups. Depending on the staining target, ImageJ‐based quantification included mean fluorescence intensityor positive‐area fraction, as appropriate. Three independent animals from each group were analyzed at each indicated time point (n = 3).

### 4.14 Western Blot Analysis of Wound Tissues

On day 7, wound tissues were collected from the infected control, Low‐Gel, Up‐Gel@miR‐sEVs, and Bil‐Gel@miR‐sEVs groups. Tissue samples were homogenized in ice‐cold RIPA lysis buffer supplemented with protease and phosphatase inhibitors, after which total protein concentrations were determined using the BCA assay.

Western blotting was conducted according to the procedure described above. IRAK1, phosphorylated NF‐κB p65 (p‐p65), total NF‐κB p65, and GAPDH were examined. IRAK1 abundance was normalized to GAPDH, whereas NF‐κB activation was evaluated from the p‐p65/total p65 ratio. Finally, each mouse represented one independent biological replicate, and three animals were analyzed for each group (n = 3).

### 4.15 RNA Sequencing and Transcriptomic Analysis

On day 14, wound tissues were collected from the infected control group (group I) and Bil‐Gel@miR‐sEVs group (group VI), with three independent mice included in each group (n = 3). Each individual mouse represented one independent biological replicate. Total RNA was extracted using TRIzol reagent (Vazyme), followed by treatment with DNase I to remove residual genomic DNA.

RNA library preparation, sequencing, and initial bioinformatic processing were conducted by Shanghai Personal Biotechnology Co., Ltd. (Shanghai, China). Libraries were sequenced using an Illumina NovaSeq X Plus platform, and differential‐expression analysis was subsequently performed with DESeq2. According to the predefined screening criteria, genes with |log_2_ fold change| ≥ 1 and *p* < 0.05 were retained.

Functional enrichment was evaluated using GO and KEGG over‐representation analyses in combination with GSEA. For GSEA, gene sets with an absolute |NES| > 1 and *p* < 0.05 were retained for subsequent interpretation. Heatmaps were generated in R using the pheatmap package.

### 4.16 Statistical Analysis

Quantitative data are presented as mean ± standard deviation (SD). Unless otherwise specified, each experiment was performed with at least three independent replicates (n ≥ 3). Normality and homogeneity of variance were assessed using the Shapiro–Wilk and Levene tests, respectively, where applicable. Two‐group comparisons were performed using two‐sided unpaired Student's t‐tests or Welch's t‐tests when variances were unequal. Multiple‐group comparisons were performed using one‐way analysis of variance (ANOVA)followed by Tukey's post hoc test. Time‐course data were analyzed using two‐way ANOVA, with repeated‐measures models, Greenhouse–Geisser correction, and Šídák multiple‐comparisons test applied where appropriate. Statistical analyses were performed using IBM SPSS Statistics 27.0, with R used for transcriptomic analyses. The exact sample size and statistical test for each experiment are specified in the corresponding figure legends. A value of *p* < 0.05 was considered statistically significant (^*^ *p* < 0.05, ^**^ *p* < 0.01, ^***^ *p* < 0.001; ns, not significant).

## Author Contributions

**Gaofei Zhang**: Conceptualization, Formal analysis, Methodology, Software, Writing – original draft, Writing – review & editing. **Boheng Zhu**: Data curation, Formal analysis, Methodology, Software. **Shihao Deng**: Formal analysis, Methodology, Software. **Pan Du**: Data curation, Methodology. **Jun Pu**: Formal analysis, Resources. **Xin Wang**: Software, Validation. **Shuqian Dou**: Methodology. **Yifei Wang**: Methodology. **Wenjun Liu**: Funding acquisition, Investigation, Project administration, Supervision.

## Statement on Use of Generative AI

During the preparation of this manuscript, the authors used ChatGPT (OpenAI; GPT‐5.6 Sol) to assist in the conceptualization and visual refinement of schematic illustrations. BioRender.com was used for schematic assembly. All scientific content and final illustrations were reviewed and approved by the authors, who take full responsibility for the content of the manuscript. Generative AI was not used to generate or modify experimental data, microscopy images, western blots, quantitative graphs, or statistical analyses.

## Ethics Statement

Animal experiments were approved by the Animal Ethics Committee of the Second Affiliated Hospital of Kunming Medical University (approval no: kyfey2026055) and conducted in accordance with the approved protocol.

## Conflicts of Interest

The authors declare no conflicts of interest.

## Supporting information




**Supporting File**: advs78491‐sup‐0001‐SuppMat.docx.

## Data Availability

The sequencing data generated in this study have been deposited in the NCBI Sequence Read Archive (SRA). The small‐RNA sequencing data from hUC‐MSC‐derived sEVs have been deposited under BioProject accession number PRJNA1528928, the RNA‐sequencing data from LPS‐stimulated THP‐1‐derived macrophages under PRJNA1530392, and the day‐14 wound‐tissue RNA‐sequencing data under PRJNA1528919. Other data supporting the findings of this study are available within the article and its Supporting Information, with additional raw data available from the corresponding author upon reasonable request.

## References

[1] D. G. Armstrong , A. J. M. Boulton , and S. A. Bus , “Diabetic Foot Ulcers and Their Recurrence,” New England Journal of Medicine 376, no. 24 (2017): 2367–2375, 10.1056/NEJMra1615439. 28614678

[2] W. J. Jeffcoate , L. Vileikyte , E. J. Boyko , D. G. Armstrong , and A. J. M. Boulton , “Current Challenges and Opportunities in the Prevention and Management of Diabetic Foot Ulcers,” Diabetes Care 41, no. 4 (2018): 645–652, 10.2337/dc17-1836. 29559450

[3] Y. Guan , H. Niu , Z. Liu , et al., “Sustained Oxygenation Accelerates Diabetic Wound Healing by Promoting Epithelialization and Angiogenesis and Decreasing Inflammation,” Science Advances 7, no. 35 (2021): abj0153, 10.1126/sciadv.abj0153. PMC8397271 34452918

[4] G. C. Gurtner , S. Werner , Y. Barrandon , and M. T. Longaker , “Wound Repair and Regeneration,” Nature 453, no. 7193 (2008): 314–321, 10.1038/nature07039. 18480812

[5] S. A. Eming , P. Martin , and M. Tomic‐Canic , “Wound Repair and Regeneration: Mechanisms, Signaling, and Translation,” Science Translational Medicine 6, no. 265 (2014): 265sr6, 10.1126/scitranslmed.3009337. PMC4973620 25473038

[6] G. A. James , E. Swogger , R. Wolcott , et al., “Biofilms in Chronic Wounds,” Wound Repair and Regeneration 16, no. 1 (2008): 37–44, 10.1111/j.1524-475X.2007.00321.x. 18086294

[7] D. Pranantyo , C. K. Yeo , Y. Wu , et al., “Hydrogel Dressings With Intrinsic Antibiofilm and Antioxidative Dual Functionalities Accelerate Infected Diabetic Wound Healing,” Nature Communications 15, no. 1 (2024): 954, 10.1038/s41467-024-44968-y. PMC10830466 38296937

[8] C. Tu , H. Lu , T. Zhou , et al., “Promoting the Healing of Infected Diabetic Wound by an Anti‐Bacterial and Nano‐Enzyme‐Containing Hydrogel With Inflammation‐Suppressing, ROS‐Scavenging, Oxygen and Nitric Oxide‐Generating Properties,” Biomaterials 286 (2022): 121597, 10.1016/j.biomaterials.2022.121597. 35688112

[9] L. Deng , C. Du , P. Song , et al., “The Role of Oxidative Stress and Antioxidants in Diabetic Wound Healing,” Oxidative Medicine and Cellular Longevity 2021 (2021): 8852759, 10.1155/2021/8852759. PMC7884160 33628388

[10] H. Zhang , Z. Yan , J. Zhu , et al., “Extracellular Mitochondrial‐Derived Vesicles Affect the Progression of Diabetic Foot Ulcer by Regulating Oxidative Stress and Mitochondrial Dysfunction,” Advanced Science 12, no. 10 (2025): 2407574, 10.1002/advs.202407574. PMC11904950 39835574

[11] H. Li , S. Wei , Q. Ling , et al., “Nanozyme‐Reinforced Hydrogel Spray as a Reactive Oxygen Species‐Driven Oxygenator to Accelerate Diabetic Wound Healing,” Advanced Materials 37, no. 34 (2025): 2504829, 10.1002/adma.202504829. 40492891

[12] H. Yang , D. Lv , S. Qu , et al., “A ROS‐Responsive Lipid Nanoparticles Release Multifunctional Hydrogel Based on Microenvironment Regulation Promotes Infected Diabetic Wound Healing,” Advanced Science 11, no. 43 (2024): 2403219, 10.1002/advs.202403219. PMC11578374 39308241

[13] Y. Liu , X. Yang , K. Wu , et al., “Skin‐Inspired and Self‐Regulated Hydrophobic Hydrogel for Diabetic Wound Therapy,” Advanced Materials 37, no. 16 (2025): 2414989, 10.1002/adma.202414989. 40059610

[14] Z. Xu , G. Liu , P. Liu , et al., “Hyaluronic Acid‐Based Glucose‐Responsive Antioxidant Hydrogel Platform for Enhanced Diabetic Wound Repair,” Acta Biomaterialia 147 (2022): 147–157, 10.1016/j.actbio.2022.05.047. 35649507

[15] W. Zhang , K. Zha , Y. Xiong , et al., “Glucose‐Responsive, Antioxidative HA‐PBA‐FA/EN106 Hydrogel Enhanced Diabetic Wound Healing Through Modulation of FEM1b‐FNIP1 Axis and Promoting Angiogenesis,” Bioactive Materials 30 (2023): 29–45, 10.1016/j.bioactmat.2023.07.006. PMC10382778 37521275

[16] T. A. Wynn and K. M. Vannella , “Macrophages in Tissue Repair, Regeneration, and Fibrosis,” Immunity 44, no. 3 (2016): 450–462, 10.1016/j.immuni.2016.02.015. PMC4794754 26982353

[17] A. E. Louiselle , S. M. Niemiec , C. Zgheib , and K. W. Liechty , “Macrophage Polarization and Diabetic Wound Healing,” Translational Research 236 (2021): 109–116, 10.1016/j.trsl.2021.05.006. 34089902

[18] M. Sharifiaghdam , E. Shaabani , R. Faridi‐Majidi , S. C. De Smedt , K. Braeckmans , and J. C. Fraire , “Macrophages as a Therapeutic Target to Promote Diabetic Wound Healing,” Molecular Therapy 30, no. 9 (2022): 2891–2908, 10.1016/j.ymthe.2022.07.016. PMC9482022 35918892

[19] Y.‐J. Fu , Y.‐F. Shi , L.‐Y. Wang , et al., “All‐Natural Immunomodulatory Bioadhesive Hydrogel Promotes Angiogenesis and Diabetic Wound Healing by Regulating Macrophage Heterogeneity,” Advanced Science 10, no. 13 (2023): 2206771, 10.1002/advs.202206771. PMC10161050 36862027

[20] S. Butenko , R. R. Nagalla , C. F. Guerrero‐Juarez , et al., “Hydrogel Crosslinking Modulates Macrophages, Fibroblasts, and Their Communication, During Wound Healing,” Nature Communications 15, no. 1 (2024): 6820, 10.1038/s41467-024-50072-y. PMC11315930 39122702

[21] Z. Liu , X. Bian , L. Luo , et al., “Spatiotemporal Single‐Cell Roadmap of Human Skin Wound Healing,” Cell Stem Cell 32, no. 3 (2025): 479–498.e8, 10.1016/j.stem.2024.11.013. 39729995

[22] K. D. Taganov , M. P. Boldin , K.‐J. Chang , and D. Baltimore , “NF‐Κb‐Dependent Induction of Microrna Mir‐146, an Inhibitor Targeted to Signaling Proteins of Innate Immune Responses,” Proceedings of the National Academy of Sciences 103, no. 33 (2006): 12481–12486, 10.1073/pnas.0605298103. PMC1567904 16885212

[23] M. P. Boldin , K. D. Taganov , D. S. Rao , et al., “Mir‐146a is a Significant Brake on Autoimmunity, Myeloproliferation, and Cancer in Mice,” Journal of Experimental Medicine 208, no. 6 (2011): 1189–1201, 10.1084/jem.20101823. PMC3173243 21555486

[24] Q. Li , W. Hu , Q. Huang , et al., “Mir146a‐Loaded Engineered Exosomes Released From Silk Fibroin Patch Promote Diabetic Wound Healing by Targeting IRAK1,” Signal Transduction and Targeted Therapy 8, no. 1 (2023): 62, 10.1038/s41392-022-01263-w. PMC9922687 36775818

[25] H.‐R. Chen , H.‐S. Huang , S.‐Y. Zeng , et al., “Mir‐146a‐5p Targets IRAK1/TRAF6 to Promote Bacillus Calmette‐Guérin Survival by Exosome‐Mediated Autocrine Actions,” Molecular Immunology 185 (2025): 105–115, 10.1016/j.molimm.2025.07.012. 40730095

[26] X. Yang , S. Hong , T. Yan , et al., “Mir‐146a Engineered Extracellular Vesicles Derived From Mesenchymal Stromal Cells More Potently Attenuate Ischaemia–Reperfusion Injury in Lung Transplantation,” Clinical and Translational Medicine 15, no. 4 (2025): 70298, 10.1002/ctm2.70298. PMC11975614 40195092

[27] R. Kalluri and V. S. LeBleu , “The Biology, Function, and Biomedical Applications of Exosomes,” Science 367, no. 6478 (2020): aau6977, 10.1126/science.aau6977. PMC7717626 32029601

[28] Y. Li , Z. Zhu , S. Li , et al., “Exosomes: Compositions, Biogenesis, and Mechanisms in Diabetic Wound Healing,” Journal of Nanobiotechnology 22, no. 1 (2024): 398, 10.1186/s12951-024-02684-1. PMC11225131 38970103

[29] S. Rui , L. Dai , X. Zhang , et al., “Exosomal Mirna‐26b‐5p From PRP Suppresses Nets by Targeting MMP‐8 to Promote Diabetic Wound Healing,” Journal of Controlled Release 372 (2024): 221–233, 10.1016/j.jconrel.2024.06.050. 38909697

[30] F. Back , A. Barras , A. Nyam‐Erdene , et al., “Platelet Extracellular Vesicles Loaded Gelatine Hydrogels for Wound Care,” Advanced Healthcare Materials 14, no. 1 (2025): 2401914, 10.1002/adhm.202401914. PMC11694086 39449544

[31] Q. Wei , J. Su , S. Meng , et al., “Mir‐17‐5p‐Engineered Sevs Encapsulated in Gelma Hydrogel Facilitated Diabetic Wound Healing by Targeting PTEN and P21,” Advanced Science 11, no. 13 (2024): 2307761, 10.1002/advs.202307761. PMC10987139 38286650

[32] Y. Song , Y. You , X. Xu , et al., “Adipose‐Derived Mesenchymal Stem Cell‐Derived Exosomes Biopotentiated Extracellular Matrix Hydrogels Accelerate Diabetic Wound Healing and Skin Regeneration,” Advanced Science 10, no. 30 (2023): 2304023, 10.1002/advs.202304023. PMC10602544 37712174

[33] Y. Yang , J. Zhang , S. Wu , et al., “Exosome/Antimicrobial Peptide Laden Hydrogel Wound Dressings Promote Scarless Wound Healing Through Mir‐21‐5p‐Mediated Multiple Functions,” Biomaterials 308 (2024): 122558, 10.1016/j.biomaterials.2024.122558. 38581764

[34] N. Hu , Z. Cai , X. Jiang , et al., “Hypoxia‐Pretreated ADSC‐Derived Exosome‐Embedded Hydrogels Promote Angiogenesis and Accelerate Diabetic Wound Healing,” Acta Biomaterialia 157 (2023): 175–186, 10.1016/j.actbio.2022.11.057. 36503078

[35] Y. Zhang , M. Li , Y. Wang , et al., “Exosome/Metformin‐Loaded Self‐Healing Conductive Hydrogel Rescues Microvascular Dysfunction and Promotes Chronic Diabetic Wound Healing by Inhibiting Mitochondrial Fission,” Bioactive Materials 26 (2023): 323–336, 10.1016/j.bioactmat.2023.01.020. PMC10027478 36950152

[36] X. Han , C. Saengow , L. Ju , W. Ren , R. H. Ewoldt , and J. Irudayaraj , “Exosome‐Coated Oxygen Nanobubble‐Laden Hydrogel Augments Intracellular Delivery of Exosomes for Enhanced Wound Healing,” Nature Communications 15, no. 1 (2024): 3435, 10.1038/s41467-024-47696-5. PMC11039765 38653959

[37] G. Xu , J. Jin , Z. Fu , et al., “Extracellular Vesicle‐Based Drug Overview: Research Landscape, Quality Control and Nonclinical Evaluation Strategies,” Signal Transduction and Targeted Therapy 10, no. 1 (2025): 255, 10.1038/s41392-025-02312-w. PMC12350758 40804047

[38] J. A. Korchak , E. F. Wiest , and A. C. Zubair , “How Do We Assess Batch‐To‐Batch Consistency Between Extracellular Vesicle Products?,” Transfusion 63, no. 2 (2023): 279–287, 10.1111/trf.17156. 36329638

[39] J.‐H. Park , M.‐Y. Ahn , Y.‐C. Kim , et al., “In Vitro and In Vivo Antimicrobial Effect of Photodynamic Therapy Using a Highly Pure Chlorin E6 Against Staphylococcus Aureus Xen29,” Biological and Pharmaceutical Bulletin 35, no. 4 (2012): 509–514, 10.1248/bpb.35.509. 22466554

[40] M. Li , S. Zhou , Q. Yu , et al., “A Sprayable TQ/Ce6@SAB/F‐Gel for Accelerating Wound Healing Via Hypoxia‐Tolerant Photodynamic Therapy and Immune–Metabolic Pathway,” Biomaterials 325 (2026): 123602, 10.1016/j.biomaterials.2025.123602. 40782448

[41] X. Wang , J. Ding , X. Chen , et al., “Light‐Activated Nanoclusters With Tunable ROS for Wound Infection Treatment,” Bioactive Materials 41 (2024): 385–399, 10.1016/j.bioactmat.2024.07.009. PMC11342113 39184828

[42] Z. Wang , S. Zeng , Y. Hao , et al., “Gram‐Negative Bacteria Recognition and Photodynamic Elimination by Zn‐DPA Based Sensitizers,” Biomaterials 308 (2024): 122571, 10.1016/j.biomaterials.2024.122571. 38636132

[43] T. Wei , T. Pan , X. Peng , et al., “Janus Liposozyme for the Modulation of Redox and Immune Homeostasis in Infected Diabetic Wounds,” Nature Nanotechnology 19, no. 8 (2024): 1178–1189, 10.1038/s41565-024-01660-y. 38740936

[44] H. Li , H. Xie , J. Zhang , et al., “Hydrogel‐Based Sequential Photodynamic Therapy Promotes Wound Healing by Targeting Wound Infection and Inflammation,” Nano Letters 25, no. 32 (2025): 12107–12117, 10.1021/acs.nanolett.5c00014. 40762315

[45] A. Maleki , J. He , S. Bochani , V. Nosrati , M.‐A. Shahbazi , and B. Guo , “Multifunctional Photoactive Hydrogels for Wound Healing Acceleration,” ACS Nano 15, no. 12 (2021): 18895–18930, 10.1021/acsnano.1c08334. 34870413

[46] S. Cheng , H. Wang , X. Pan , et al., “Dendritic Hydrogels With Robust Inherent Antibacterial Properties for Promoting Bacteria‐Infected Wound Healing,” ACS Applied Materials & Interfaces 14, no. 9 (2022): 11144–11155, 10.1021/acsami.1c25014. 35195389

[47] X. Qi , Y. Li , Y. Xiang , et al., “Hyperthermia‐Enhanced Immunoregulation Hydrogel for Oxygenation and ROS Neutralization in Diabetic Foot Ulcers,” Cell Biomaterials 1, no. 3 (2025): 100020, 10.1016/j.celbio.2025.100020.

[48] Q. Zeng , X. Qi , G. Shi , M. Zhang , and H. Haick , “Wound Dressing: From Nanomaterials to Diagnostic Dressings and Healing Evaluations,” ACS Nano 16, no. 2 (2022): 1708–1733, 10.1021/acsnano.1c08411. 35050565

[49] X. Qi , E. Cai , Y. Xiang , et al., “An Immunomodulatory Hydrogel by Hyperthermia‐Assisted Self‐Cascade Glucose Depletion and ROS Scavenging for Diabetic Foot Ulcer Wound Therapeutics,” Advanced Materials 35, no. 48 (2023): 2306632, 10.1002/adma.202306632. 37803944

[50] F. Dai , J. Zhang , F. Chen , et al., “A Multi‐Responsive Hydrogel Combined With Mild Heat Stimulation Promotes Diabetic Wound Healing by Regulating Inflammatory and Enhancing Angiogenesis,” Advanced Science 11, no. 46 (2024): 2408783, 10.1002/advs.202408783. PMC11633493 39435670

[51] F. He , P. Xu , Z. Zhu , et al., “Inflammation‐Responsive Hydrogel Accelerates Diabetic Wound Healing Through Immunoregulation and Enhanced Angiogenesis,” Advanced Healthcare Materials 14, no. 5 (2025): 2400150, 10.1002/adhm.202400150. 38663034

[52] H. Yu , J. Zhang , L. Yang , et al., “MSC‐Derived Exosomes Injectable Hyaluronic Acid Hydrogel for Enhanced Chronic Wound Healing,” Journal of Controlled Release 385 (2025): 113985, 10.1016/j.jconrel.2025.113985. 40581219

[53] G. Theocharidis , B. E. Thomas , D. Sarkar , et al., “Single Cell Transcriptomic Landscape of Diabetic Foot Ulcers,” Nature Communications 13, no. 1 (2022): 181, 10.1038/s41467-021-27801-8. PMC8748704 35013299

[54] Y. Dong , M. Wang , Q. Wang , X. Cao , P. Chen , and Z. Gong , “Single‐Cell RNA ‐Seq in Diabetic Foot Ulcer Wound Healing,” Wound Repair and Regeneration 32, no. 6 (2024): 880–889, 10.1111/wrr.13218. PMC11584366 39264020

[55] H. Du , S. Li , J. Lu , et al., “Single‐Cell RNA‐Seq and Bulk‐Seq Identify RAB17 as a Potential Regulator of Angiogenesis by Human Dermal Microvascular Endothelial Cells in Diabetic Foot Ulcers,” Burns & Trauma 11 (2023): tkad020, 10.1093/burnst/tkad020. PMC10440157 37605780

