# Supplementary material for: Stage‐Adaptive Bilayer Hydrogel Coordinates Photodynamic Antibacterial Therapy with Engineered Extracellular Vesicle–Mediated Immunomodulation in Infected Diabetic Wounds
Source: Adv Sci (Weinh). 2026 Nov 10:e78491. Online ahead of print. doi: 10.1002/advs.78491 (PMC13749112; doi:10.1002/advs.78491)
Supplement: Supplementary file 1 [file ADVS-9999-e78491-s001.docx]

**Supporting Information**

**Stage-Adaptive Bilayer Hydrogel Coordinates Photodynamic Antibacterial Therapy with Engineered Extracellular Vesicle–Mediated Immunomodulation in Infected Diabetic Wounds**

Gaofei Zhang^a b 1^. Boheng Zhu^a b 1^. Shihao Deng^a b 1^. Pan Du^a b 1^. Jun Pu^a b^ . Xin Wang^a b^ . Shuqian Dou^a b^ . Yifei Wang^a b^ . Wenjun Liu^a b *^.

^a^The Second Affiliated Hospital of Kunming Medical University, Kunming, Yunnan, 650000, China

^b^Kunming Medical University, Kunming, Yunnan, 650500, China

^1^Gaofei Zhang, Boheng Zhu, Shihao Deng, and Pan Du contributed equally to this work.

^*^Corresponding author, Wenjun Liu.

This Supporting Information contains Supplementary Experimental Procedures, Table S1, Figures S1–S10, and Supplementary Sequence Data (Sequences S1–S2).

# ****Contents****

Supplementary Experimental Procedures

Table S1. Primer sequences used for quantitative reverse-transcription PCR (qRT-PCR)

**Figure S1.** Small-RNA sequencing and validation for miR-146a selection

**Figure S2.** Structural and macroscopic characterization of the hydrogel compartments

**Figure S3.** Intracellular ROS-associated fluorescence in HUVECs and PMA-differentiated THP-1 macrophages

**Figure S4.** CD64 expression before and after PMA-induced THP-1 differentiation

**Figure S5.** Exploratory transcriptomic analysis of LPS-stimulated PMA-differentiated THP-1 macrophages following miR-sEVs treatment

**Figure S6.** Fibroblast repair-associated responses

**Figure S7.** Histological and immunofluorescence evaluation of infected diabetic wounds

**Figure S8.** Cytocompatibility, hemocompatibility, and preliminary short-term in vivo safety

**Figure S9.** Full-length, uncropped western blot source images

**Figure S10.** Characterization of hUC-MSCs and hUC-MSC-derived sEVs for small-RNA screening

Supplementary Sequence Data. Verified construct sequences (Sequences S1–S2)

# Supplementary Experimental Procedures

### **S1. Isolation and Characterization of hUC-MSC-Derived sEVs**

Small extracellular vesicles (sEVs) were isolated from the conditioned medium of commercially sourced human umbilical cord mesenchymal stromal cells (hUC-MSCs; Cyagen Biosciences Inc., Cat. No. HUXUC-01001) by sequential centrifugation, membrane filtration, and ultracentrifugation. At passages 3–5, hUC-MSCs were maintained in Dulbecco’s modified Eagle medium (DMEM) supplemented with EV-depleted fetal bovine serum (Gibco, Thermo Fisher Scientific, USA). At 80–90% confluence, cells were treated with lipopolysaccharide (LPS; 100 ng mL⁻¹) for 48 h, while untreated cultures were maintained in parallel. sEVs collected from untreated and LPS-treated cultures were designated MSC-sEVs and LPS-MSC-sEVs, respectively.

Conditioned medium was first centrifuged at 10 000 × g for 30 min at 4 °C to remove residual cellular debris and then passed through a 0.22 μm membrane filter. The filtrate was ultracentrifuged at 100 000 × g for 3 h at 4 °C using an Optima L-100XP ultracentrifuge (Beckman Coulter, USA). The resulting sEV pellets were resuspended in sterile phosphate-buffered saline (PBS) and stored at −80 °C until further use.

sEV protein concentrations were quantified using a bicinchoninic acid (BCA) Protein Assay Kit (P0012, Beyotime Biotechnology, Shanghai, China). Particle concentration and size distribution were determined by nanoparticle tracking analysis (NTA), and vesicle morphology was examined by transmission electron microscopy (TEM) after negative staining with uranyl acetate. The EV-associated proteins CD9, CD81, and TSG101 were analyzed by western blotting, with Calnexin included as a non-EV cellular marker and hUC-MSC lysates serving as a positive control. Representative hUC-MSC morphology and multilineage differentiation, together with the characterization of MSC-sEVs and LPS-MSC-sEVs, are shown in Figure S10.

### **S2. Small-RNA Sequencing and Quantitative Reverse-Transcription PCR (qRT-PCR) Validation**

Small-RNA sequencing was performed on MSC-sEVs and LPS-MSC-sEVs prepared as described in Section S1, with three independently prepared sEV samples analyzed per group. Total RNA was extracted from purified sEV preparations using the miRNeasy Micro Kit (Qiagen, Hilden, Germany; Cat. No. 217084). Small-RNA libraries were generated from 1 μg of total RNA per sample using the NEBNext Multiplex Small RNA Library Prep Set for Illumina and sequenced on an Illumina NovaSeq X Plus platform. Library preparation, sequencing, and bioinformatic analysis were performed by Shanghai Personal Biotechnology Co., Ltd. (Shanghai, China).

Raw reads were adapter-trimmed and quality-filtered, and reads outside the 18–36 nt range were excluded. Clean reads were aligned to the human GRCh38 reference genome, and known miRNAs were annotated using miRBase release 22. miRNA abundance was normalized as transcripts per million mapped reads (TPM). Differential-expression analysis was performed using DESeq2, with |log₂ fold change| > 1 and p < 0.05 used as the screening thresholds. Each independently prepared sEV sample was treated as one biological replicate.

Selected candidate miRNAs were subsequently validated by qRT-PCR using equal amounts of total sEV RNA. Reverse transcription was performed using HiScript III RT SuperMix for qPCR (+gDNA wiper; R323-01, Vazyme Biotech Co., Ltd., Nanjing, China), followed by amplification with SupRealQ Purple Universal SYBR qPCR Master Mix (U+; Q412-02, Vazyme) on a QuantStudio 6 Flex Real-Time PCR System (Thermo Fisher Scientific, Waltham, MA, USA). Relative miRNA expression was calculated using the 2^−ΔΔCt method, with miR-22-5p as the endogenous reference. Primer sequences are listed in Table S1.

### **S3. Structural and Macroscopic Characterization of the Hydrogels**

The internal microstructures of the hydrogel compartments were examined by scanning electron microscopy (SEM) using freeze-dried samples. Apparent pore sizes were quantified from the corresponding SEM images using ImageJ. Because these measurements were obtained from dehydrated specimens, the reported values represent apparent pore dimensions rather than hydrated network mesh sizes.

Swelling behavior was evaluated by incubating independently prepared Low-Gel, Up-Gel, and Bil-Gel samples at 37 °C and recording changes in sample mass at the indicated time points. Relative swelling ratios were calculated from the measured masses, with five independently prepared samples analyzed for each formulation.

To assess ROS-responsive degradation behavior, Low-Gel and Up-Gel samples were incubated in PBS or PBS containing the indicated concentrations of H₂O₂ at 37 °C. Samples were collected at predetermined time points, and the remaining wet mass was recorded relative to the initial wet mass. Three independently prepared samples were analyzed for each condition.

Macroscopic recovery, flexibility, deformability, and moldability were evaluated during manual handling and documented photographically. Recovery of Low-Gel after mechanical disruption was assessed by completely cutting the hydrogel and subsequently bringing the freshly exposed surfaces back into contact without additional adhesive or external crosslinking. Flexibility and deformability were examined by manual bending, stretching, and compression, whereas moldability was demonstrated by shaping the hydrogels into defined macroscopic forms.

Wet-tissue adhesion of Bil-Gel was additionally evaluated qualitatively by placing the hydrogel directly onto freshly excised moist liver and lung tissues and documenting its retention during gentle manipulation. Quantitative wet-tissue adhesion measurements using freshly excised porcine skin were performed as described in the main Experimental Section.

The corresponding structural, swelling, degradation, macroscopic handling, and qualitative wet-tissue adhesion results are presented in Figure S2.

## **S4. Supplementary Details of PKH67 Labeling, Macrophage Uptake, Flow Cytometry, and IRAK1/NF-κB Signaling Analysis**

Engineered miR-sEVs were fluorescently labeled with PKH67 according to the manufacturer’s instructions. Unincorporated dye was removed using an exosome free-dye removal spin-column kit (Cat. No. E0015-5T, Mele Biotech, Hubei, China). Briefly, the labeled sEV suspension was loaded onto a prewashed spin column, incubated at room temperature for 5 min, and centrifuged at 1500 × g for 1 min at 4 °C. Purified PKH67-labeled miR-sEVs were collected from the flow-through and incubated overnight at 37 °C with PMA-differentiated THP-1 macrophages with or without prior CD64 blockade. Cellular uptake was examined by confocal laser scanning microscopy. For CD64-blockade experiments, macrophages were preincubated with anti-human CD64 antibody (clone 10.1; 10 μg mL⁻¹) for 1 h before exposure to miR-sEVs at a final protein concentration of 100 μg mL⁻¹. Signaling and surface-marker analyses were performed after 24 h of miR-sEV treatment.

For macrophage surface-marker analysis, treated cells were stained with PE-conjugated anti-human CD86 antibody (clone IT2.2, BioLegend, Cat. No. 305405) and APC-conjugated anti-human CD206 antibody (clone 15-2, BioLegend, Cat. No. 321110) according to the manufacturer’s instructions. Data were acquired using a FACSCanto II flow cytometer (BD Biosciences, USA) and analyzed using FlowJo v10.0.7r2 (Tree Star, Inc., Ashland, OR, USA). Cellular debris was excluded on the basis of forward- and side-scatter characteristics, followed by singlet gating to exclude cell aggregates. The percentages of CD86-positive and CD206-positive cells, together with the CD86-positive/CD206-positive ratio, were calculated.

For western blot analysis, total cellular protein was extracted using radioimmunoprecipitation assay (RIPA) lysis buffer supplemented with protease and phosphatase inhibitors and quantified by bicinchoninic acid assay. Equal amounts of protein (30 μg per lane) were separated by 10%–15% gradient sodium dodecyl sulfate–polyacrylamide gel electrophoresis (SDS-PAGE) and transferred onto 0.22 μm polyvinylidene fluoride (PVDF) membranes by wet electrotransfer. Membranes were blocked with 5% bovine serum albumin (BSA) in Tris-buffered saline containing Tween 20 (TBST) for 1 h and incubated overnight at 4 °C with primary antibodies against IRAK1, phosphorylated NF-κB p65, total NF-κB p65, and GAPDH. After washing, the membranes were incubated with the corresponding horseradish peroxidase (HRP)-conjugated secondary antibodies for 1 h at room temperature. Protein bands were visualized by enhanced chemiluminescence and quantified using ImageJ. IRAK1 was normalized to GAPDH, whereas phosphorylated p65 was normalized to total p65. Full-length, uncropped western blot source images are provided in Figure S9.

Intracellular miR-146a and the mRNA expression levels of TRAF6, RELA, and NFKBIA were measured by qRT-PCR using the reverse-transcription and amplification reagents described in Section S2. GAPDH served as the endogenous reference for mRNA analysis, whereas U6 was used for normalization of cellular miR-146a. Relative expression was calculated using the 2^−ΔΔCt method. Primer sequences are provided in Table S1. Three independent cell cultures were analyzed per group.

### **S5. Transcriptomic Analysis of LPS-Stimulated Macrophages following miR-sEV Treatment**

Transcriptomic profiling was performed in PMA-differentiated THP-1 macrophages to assess transcriptome-wide changes associated with miR-sEV treatment under inflammatory conditions. Macrophages were stimulated with LPS (100 ng mL⁻¹) for 24 h and then treated with miR-sEVs (100 μg mL⁻¹) for a further 24 h. LPS-stimulated macrophages without miR-sEV treatment served as controls. Three independent cell cultures were analyzed per group.

Total RNA was isolated from each independent culture, and RNA concentration, purity, and integrity were evaluated before library preparation. Samples that met the required quality criteria were used for sequencing. Raw reads were quality-filtered to remove adapter-containing and low-quality sequences, and gene-level expression values were obtained for downstream analyses.

Principal-component analysis (PCA) was used to examine global variation among the transcriptomic profiles. Differential-expression analysis was performed using DESeq2. Genes with a fold change ≥ 1.2 or ≤ 0.83 and nominal p < 0.05 were retained according to the applied screening criteria and displayed in volcano plots.

Gene Ontology (GO) and Kyoto Encyclopedia of Genes and Genomes (KEGG) over-representation analyses were used for functional annotation of the screened genes. Gene Set Enrichment Analysis (GSEA) was performed separately using the ranked whole-gene expression dataset. Both nominal and multiple-testing-adjusted p values were considered when interpreting the GSEA results. Gene sets that did not remain significant after multiple-testing correction were treated as exploratory findings.

Heatmaps of selected inflammation- and macrophage-associated genes were generated using the pheatmap package in R. Expression values were standardized within each gene and displayed as row-scaled Z-scores.

The corresponding macrophage transcriptomic analyses are shown in Figure S5, and the related GO enrichment results are presented in Figure 6B of the main manuscript.

## **S6. Cytocompatibility, Hemocompatibility, and Preliminary Short-Term In Vivo Safety**

## L929 fibroblasts were seeded in the lower chambers of a Transwell coculture system at 5 × 10³ cells per well, and equal amounts of Low-Gel, Up-Gel, or Bil-Gel were placed in the upper inserts. Cells cultured without hydrogel exposure served as controls. Human umbilical vein endothelial cells (HUVECs) were evaluated under the same conditions. Cellular metabolic activity was assessed on days 1, 2, and 3 using a Cell Counting Kit-8 (CCK-8) assay, with absorbance measured at 450 nm using a Synergy H1 multimode microplate reader (BioTek, Winooski, VT, USA).

For live/dead staining, L929 fibroblasts and HUVECs were evaluated on days 1, 2, and 3. Cells were incubated with calcein-AM and propidium iodide in PBS for 30 min in the dark and imaged using an IX83 inverted fluorescence microscope (Olympus, Japan). Cytoskeletal organization and cell spreading were examined after 24 h of indirect coculture. L929 fibroblasts were seeded at 5 × 10⁴ cells per well, and HUVECs were processed under the same conditions. Cells were fixed with 4% paraformaldehyde for 15 min, stained with TRITC-phalloidin (1:200), and counterstained with DAPI (1 μg mL⁻¹). Confocal images and three-dimensional fluorescence reconstructions were acquired using a TCS SP8 laser-scanning confocal microscope (Leica Microsystems). Three randomly selected fields were analyzed for each biological replicate using Fiji/ImageJ, and the resulting measurements were averaged to obtain a single replicate value. Three independent biological replicates were analyzed per group.

Hemocompatibility was assessed using fresh anticoagulated goat blood. Hydrogel extracts were prepared by immersing equal amounts of Low-Gel, Up-Gel, or Bil-Gel in 1 mL of sterile saline (0.9% NaCl) at 37 °C for 12 h. Anticoagulated whole blood (20 μL) was added to each extract, followed by incubation at 37 °C with orbital shaking at 120 rpm for 2 h. The samples were then centrifuged at 200 × g for 10 min, and the absorbance of the supernatants was measured at 545 nm using a Synergy H1 multimode microplate reader. Deionized water and saline containing the same volume of blood served as the positive and negative controls, respectively.

The hemolysis rate was calculated as follows:

$$Hemolysis rate (\%)=\frac{A_{\mathrm{sample}}-A_{\mathrm{negative}}}{A_{\mathrm{positive}}-A_{\mathrm{negative}}}\times100\%$$

where $A_{\mathrm{sample}}$, $A_{\mathrm{negative}}$, and $A_{\mathrm{positive}}$ represent the absorbance values of the test sample, negative control, and positive control, respectively.

For short-term in vivo safety evaluation, 0.5 g of Low-Gel, Up-Gel, or Bil-Gel was surgically implanted into the subcutaneous tissue of male C57BL/6J mice aged 8–10 weeks and weighing 25–27 g. A corresponding control group was included for comparison. Ten days after implantation, the mice were euthanized, and the heart, liver, spleen, lungs, and kidneys were collected.Tissues were fixed in 4% paraformaldehyde, paraffin-embedded, sectioned, and stained with hematoxylin and eosin (H&E) for histological examination. These procedures were conducted under the same animal-ethics approval as the infected diabetic wound-healing study.

### The corresponding cytocompatibility, hemocompatibility, and preliminary short-term in vivo safety results are shown in Figure S8.

### **S7. Wound-Tissue RNA Sequencing and Transcriptomic Analysis**

For the day-14 wound-tissue transcriptomic analysis, RNA concentration, purity, and integrity were evaluated before library preparation, and only samples meeting the required quality criteria were used for sequencing. Raw reads were quality-filtered to remove adapter-containing and low-quality sequences. The resulting clean reads were aligned to the Mus musculus GRCm39 reference genome obtained from Ensembl, and gene-level read counts were generated for downstream analyses.

Gene Set Enrichment Analysis (GSEA) was performed using the ranked whole-gene expression dataset. Gene sets with |NES| > 1 and p < 0.05 were retained for interpretation. Adjusted p values were additionally reported for the principal GSEA results shown in the main manuscript. Heatmaps of representative genes were generated using the pheatmap package in R. Expression values were standardized within each gene and displayed as row-scaled Z-scores.

## Table S1. Primer sequences used for qRT-PCR.

| **Target** | **Species** | **Forward primer（5′–3′）** | **Reverse primer（5′–3′）** |
| --- | --- | --- | --- |
| **Mmp13** | Mouse | GATGACCTGTCTGAGGAAGACC | GCATTTCTCGGAGCCTGTCAAC |
| **Reg1** | Mouse | AAGGAGAGTGGCACTACAGACG | CGATTGGAACTGTTAGGAGACCC |
| **Adam8** | Mouse | TGCCAACGTGACACTGGAGAAC | GCAGACACCTTAGCCAGTCCAA |
| **Krt6a** | Mouse | GGACATTGCTCAGAGAAGTCGG | CAATCTCCTGCTTGGTGTTGCG |
| **Cdkn2a** | Mouse | TGTTGAGGCTAGAGAGGATCTTG | CGAATCTGCACCGTAGTTGAGC |
| **Epgn** | Mouse | GAGCGAAGAAGCAGAGGTGATC | GGTCTTCCAGACAAGGATGAGAG |
| **Col6a5** | Mouse | CAAACATGACACGGATCATCA | GGAACTGTCTTATCAACGTGGT |
| **Gapdh** | Mouse | CATCACTGCCACCCAGAAGACTG | ATGCCAGTGAGCTTCCCGTTCAG |
| IRAK1 | Human | TCAGAACGGCTTCTACTGCCTG | TACCCAGAAGGATGTCCAGTCG |
| TRAF6 | Human | CAATGCCAGCGTCCCTTCCAAA | CCAAAGGACAGTTCTGGTCATGG |
| RELA | Human | TGAACCGAAACTCTGGCAGCTG | CATCAGCTTGCGAAAAGGAGCC |
| NFKBIA | Human | TCCACTCCATCCTGAAGGCTAC | CAAGGACACCAAAAGCTCCACG |
| GAPDH | Human | GTCTCCTCTGACTTCAACAGCG | ACCACCCTGTTGCTGTAGCCAA |
| hsa-miR-146a-5p | Human | TGAGAACTGAATTCCATGGGTT | GAACATGTCTGCGTATCTC |
| hsa-miR-1246 | Human | AATGGATTTTTGGAGCAGG | GAACATGTCTGCGTATCTC |
| hsa-miR-1290 | Human | TGGATTTTTGGATCAGGGA | GAACATGTCTGCGTATCTC |
| hsa-let-7f-5p | Human | TGAGGTAGTAGATTGTATAGTT | GAACATGTCTGCGTATCTC |
| U6 snRNA | Human | CTCGCTTCGGCAGCACA | TTGCGTGTCATCCTTGCG |
| hsa-miR-22-5p | Human | GTTCTTCAGTGGCAAGC | GAACATGTCTGCGTATCTC |

**Figure S1. Small-RNA sequencing and validation for miR-146a selection.** A) Schematic workflow comparing sEVs derived from untreated and LPS-preconditioned hUC-MSCs. B) Venn analysis of detected miRNAs. C) Principal-component analysis (PCA). D) Differential-expression profile of miRNAs between MSC-sEVs and LPS-MSC-sEVs. E) Heatmap of representative differentially expressed miRNAs. F) Relative abundance of selected candidate miRNAs in the sequencing dataset. G) qRT-PCR validation of selected candidate miRNAs. Small-RNA sequencing was performed on three independently prepared sEV samples per group. Differential expression was analyzed using DESeq2, with |log₂ fold change| > 1 and p < 0.05 as the screening criteria. qRT-PCR data are presented as mean ± SD (n = 3 independent sEV preparations per group). Statistical comparisons were performed on ΔCt values using two-sided unpaired Student’s t-tests. **p < 0.05*, ***p < 0.01*, ****p < 0.001*; ns, not significant. Schematic panel A was created using BioRender.com.

**Figure S2. Supplementary structural and macroscopic characterization of the hydrogel compartments.** A,B) Apparent pore-size distributions of freeze-dried Low-Gel (A) and Up-Gel (B). C) Time-dependent relative swelling ratios of Low-Gel, Up-Gel, and Bil-Gel at 37 °C (n = 5 independently prepared samples per formulation). D,E) Time-dependent remaining wet mass of Low-Gel (D) and Up-Gel (E) in PBS or PBS containing the indicated concentrations of H₂O₂ (n = 3 independently prepared samples per condition). F) Representative macroscopic images of the cut-and-contact recovery of Low-Gel after reapposition of freshly cut surfaces, including recovery after 5 min of contact and subsequent mechanical handling. G,H) Representative images showing the flexibility and deformability of Low-Gel (G) and Up-Gel (H) during manual handling. I,J) Representative images showing the moldability of Low-Gel (I) and Up-Gel (J) into defined macroscopic shapes. K) Representative macroscopic images demonstrating qualitative adhesion of Bil-Gel to a glass vial cap, the base of a glass vial, a centrifuge tube, and freshly excised moist liver and lung tissues during gentle manipulation. Panels A and B show descriptive pore-size distributions. Quantitative data in panels C–E are presented as mean ± SD; no inferential statistical comparisons were performed. Panels F–K are qualitative and are presented descriptively.

**Figure S3. Intracellular DCFH-DA-associated fluorescence in HUVECs and PMA-differentiated THP-1 macrophages under oxidative stress.** Representative bright-field, fluorescence, and merged images of HUVECs and PMA-differentiated THP-1 macrophages simultaneously exposed to the indicated hydrogel formulations and 200 μM H₂O₂ for 3 h. Images within each cell type were acquired using identical settings. Scale bar: 50 μm. n = 3 independent cultures per group; multiple fields were examined for each culture under the same acquisition conditions.

**Figure S4. CD64 expression in THP-1 cells before and after PMA-induced differentiation.** Representative flow-cytometry plots and corresponding quantification of CD64-positive cells are shown. Data are presented as mean ± SD (n = 3 independent cultures per group). Statistical comparisons were performed using a two-sided unpaired Student’s t-test. **p < 0.05*, ***p < 0.01*, ****p < 0.001*.

**Figure S5. Exploratory transcriptomic profiling of LPS-stimulated PMA-differentiated THP-1 macrophages following miR-sEV treatment.** A) Principal-component analysis (PCA). B) Volcano plot of genes meeting the specified screening criteria. C) Gene Set Enrichment Analysis (GSEA) of the myeloid leukocyte differentiation gene set. D) Heatmap of representative macrophage-associated genes. E) KEGG pathway over-representation analysis of the screened genes. n = 3 independent cultures per group. Genes were screened using DESeq2 with a fold change ≥ 1.2 or ≤ 0.83 and nominal p < 0.05. The myeloid leukocyte differentiation gene set did not retain statistical significance after multiple-testing correction and was therefore considered exploratory.

**Figure S6. Repair-related responses of fibroblasts following the indicated treatments.** A) Representative images of EdU incorporation. B) Representative immunofluorescence images of collagen I and collagen III. C) Representative Transwell migration images. D) Representative scratch-wound images at 0, 12, and 24 h. Scale bars are indicated in the images. Corresponding quantitative analyses and statistical comparisons are presented in Figure 7L–O of the main manuscript.

**Figure S7. Supplementary histological and immunofluorescence evaluation of infected diabetic wounds.** Groups I–VI correspond to the infected control, Low-Gel, Up-Gel@miR-sEVs, Bil-Gel, Bil-Gel@sEVs, and Bil-Gel@miR-sEVs groups, respectively. A) Representative H&E-stained wound sections on day 7 at the indicated magnifications. B) Representative Masson’s trichrome-stained wound sections on day 7 at the indicated magnifications. C,D) Representative VEGF immunofluorescence images (C) and corresponding fluorescence-intensity quantification (D) on day 14. E,F) Quantification of Ki67 fluorescence intensity (E) and representative Ki67 immunofluorescence images (F) on day 14. Multiple fields or sections from each animal were averaged to yield a single biological replicate. Quantitative data are presented as mean ± SD (n = 3 independent mice per group) and were analyzed by one-way ANOVA followed by Tukey’s post hoc test. **p < 0.05*, ***p < 0.01*, ****p < 0.001*; ns, not significant. Scale bars are indicated in the images.

### **Figure S8. Cytocompatibility, hemocompatibility, and preliminary short-term in vivo safety of the hydrogel formulations.** A) Schematic illustration of the Transwell coculture setup. B) Representative morphology images of L929 fibroblasts. C) Quantification of the hemolysis rate. D,E) Representative live/dead staining images of L929 fibroblasts (D) and HUVECs (E) on days 1–3. F,G) Quantitative analyses of cellular metabolic activity in L929 fibroblasts (F) and HUVECs (G), respectively. H,I) Representative cytoskeletal staining images of L929 fibroblasts (H) and HUVECs (I), respectively. J,K) Quantification of cell-spreading area in L929 fibroblasts (J) and HUVECs (K), respectively. L) Representative H&E-stained sections of the heart, liver, spleen, lungs, and kidneys collected 10 days after subcutaneous hydrogel implantation in male C57BL/6J mice.

### For cellular assays, n = 3 independent cultures per group; for hemolysis, n = 3 independent measurements per condition; and for the implantation study, n = 3 independent mice per group. Quantitative data in panels C, F, G, J, and K are presented as mean ± SD. Panels C, J, and K were analyzed using one-way ANOVA followed by Tukey’s post hoc test. For panels F and G, statistical comparisons were performed separately at each time point using one-way ANOVA followed by Tukey’s post hoc test. Panels A, B, D, E, H, I, and L are presented descriptively. **p < 0.05*, ***p < 0.01*, ****p < 0.001*; ns, not significant. Scale bars are indicated in the corresponding images. Schematic panel A was created using BioRender.com.

**Figure S9.** Full-length, uncropped western blot source images

**Figure 1C – EV-associated proteins and Calnexin**

Original acquisition containing the three EV-associated marker membranes (CD9, CD81, and TSG101).

Calnexin original acquisition; the hUC-MSC lysate positive-control lane is retained.

**Figure S9.** Full-length, uncropped western blot source images

**Figure 3E – Day-1 released fractions**

| **CD9** | **CD81** | **TSG101** |
| --- | --- | --- |
|  |  |  |
| **Calnexin** | **GAPDH** |  |
|  |  |  |

**Figure S9.** Full-length, uncropped western blot source images

**Figure 3F – Day-14 released fractions**

| **CD9** | **CD81** | **TSG101** |
| --- | --- | --- |
|  |  |  |
| **Calnexin** | **GAPDH** |  |
|  |  |  |

**Figure S9.** Full-length, uncropped western blot source images

**Figure 6G – Macrophage IRAK1/NF-κB signaling**

| **IRAK1** | **Total NF-κB p65** |
| --- | --- |
|  |  |
| **Phosphorylated NF-κB p65** | **GAPDH** |
|  |  |

**Figure S9.** Full-length, uncropped western blot source images

**Figure 9D – Wound-tissue IRAK1/NF-κB signaling**

| **IRAK1** | **Total NF-κB p65** |
| --- | --- |
|  |  |
| **Phosphorylated NF-κB p65** | **GAPDH** |
|  |  |
|  |  |

**Figure S9.** Full-length, uncropped western blot source images

**Figure S10D – EV-associated proteins and Calnexin**

Original full-length western blot acquisitions of MSC-sEVs showing the EV-associated proteins CD9, CD81, and TSG101 together with the non-EV cellular marker Calnexin

Original full-length western blot acquisitions of LPS-MSC-sEVs showing the EV-associated proteins CD9, CD81, and TSG101 together with the non-EV cellular marker Calnexin.

**Figure S9. Full-length, uncropped western blot source images.** Full-length western blot source images corresponding to Figures 1C, 3E, 3F, 6G, 9D, and S10D are provided with the complete membrane fields and molecular-weight marker lanes retained. The corresponding proteins and source figure panels are indicated. No cropping, splicing, or selective contrast adjustment was applied to the source images; images were proportionally resized only for page layout.

# **Figure S10. Characterization of commercially sourced hUC-MSCs and hUC-MSC-derived sEVs used for small-RNA screening.** A) Representative phase-contrast images of hUC-MSCs during serial passage. B) Representative osteogenic, adipogenic, and chondrogenic differentiation of hUC-MSCs. C) Transmission electron microscopy images of MSC-sEVs and LPS-MSC-sEVs. D) Western blot analysis of the EV-associated proteins CD9, CD81, and TSG101 and the non-EV cellular protein marker Calnexin. E) Nanoparticle tracking analysis of particle-size distributions for MSC-sEVs and LPS-MSC-sEVs. Scale bars are indicated in the images.

# Supplementary Sequence Data

**Sequence S1. Nucleotide sequence of the verified coding insert of Sp-scFv(hCD64)-Gluc-C1C2-MS2.**

GTCGACTCTAGAGGATCCCCGGGTACCGGTCGCCACCATGCCGCGCCCCCGCCTGCTGGCCGCGCTGTGCGGCGCGCTGC
TCTGCGCCCCCAGCCTCCTCGTCGCCCAGGTGCAGCTTGTGGAGAGCGGTGGAGGTGTTGTGCAACCTGGCCGGTCCCTG
CGCCTGTCCTGCTCCTCGTCTGGCTTCATTTTCAGTGACAATTACATGTATTGGGTGAGACAGGCACCTGGAAAAGGTCT
TGAGTGGGTTGCAACCATTAGTGATGGTGGTAGTTACACCTACTATCCAGACAGTGTGAAGGGAAGATTTACAATATCGA
GAGACAACAGCAAGAACACATTGTTCCTGCAAATGGACAGCCTGAGACCCGAAGACACCGGGGTCTATTTTTGTGCAAGA
GGCTACTATAGGTACGAGGGGGCTATGGACTACTGGGGCCAAGGGACCCCGGTCACCGTGAGCTCAGGAGGTGGCGGCTC
CGGAGGTGGAGGCAGCGGAGGGGGCGGATCCGACATCCAGCTGACCCAGAGCCCAAGCAGCCTGAGCGCCAGCGTGGGTG
ACAGAGTGACCATCACCTGTAAGTCCAGTCAAAGTGTTTTATACAGTTCAAATCAGAAGAACTACTTGGCCTGGTACCAG
CAGAAGCCAGGTAAGGCTCCAAAGCTGCTGATCTACTGGGCATCCACTAGGGAATCTGGTGTGCCAAGCAGATTCAGCGG
TAGCGGTAGCGGTACCGACTTCACCTTCACCATCAGCAGCCTCCAGCCAGAGGACATCGCCACCTACTACTGCCATCAAT
ACCTCTCCTCGTGGACGTTCGGCCAAGGGACCAAGCTGGAGATCAAAGGTGGAGGGGGATCTGGAGGTGGTGGTAGCGGA
GGTGGGGGTTCAAAGCCCACCGAGAACAACGAAGACTTCAACATCGTGGCCGTGGCCAGCAACTTCGCGACCACGGATCT
CGATGCTGACCGCGGGAAGTTGCCCGGCAAGAAGCTGCCGCTGGAGGTGCTCAAAGAGCTGGAAGCCAATGCCCGGAAAG
CTGGCTGCACCAGGGGCTGTCTGATCTGCCTGTCCCACATCAAGTGCACGCCCAAGATGAAGAAGTTCATCCCAGGACGC
TGCCACACCTACGAAGGCGACAAAGAGTCCGCACAGGGCGGCATAGGCGAGGCGATCGTCGACATTCCTGAGATTCCTGG
GTTCAAGGACTTGGAGCCCCTGGAGCAGTTCATCGCACAGGTCGATCTGTGTGTGGACTGCACAACTGGCTGCCTCAAAG
GGCTTGCCAACGTGCAGTGTTCTGACCTGCTCAAGAAGTGGCTGCCGCAACGCTGTGCGACCTTTGCCAGCAAGATCCAG
GGCCAGGTGGACAAGATCAAGGGGGCCGGTGGTGACGCTAGCACGAAATGTGTCGAGCCACTGGGCCTGGAGAATGGGAA
CATTGCCAACTCACAGATCGCCGCCTCGTCTGTGCGTGTGACCTTCTTGGGTTTGCAGCATTGGGTCCCGGAGCTGGCCC
GCCTGAACCGCGCAGGCATGGTCAATGCCTGGACACCCAGCAGCAATGACGATAACCCCTGGATCCAGGTGAACCTGCTG
CGGAGGATGTGGGTAACAGGTGTGGTGACGCAGGGTGCCAGCCGCTTGGCCAGTCATGAGTACCTGAAGGCCTTCAAGGT
GGCCTACAGCCTTAATGGACACGAATTCGATTTCATCCATGATGTTAATAAAAAACACAAGGAGTTTGTGGGTAACTGGA
ACAAAAACGCGGTGCATGTCAACCTGTTTGAGACCCCTGTGGAGGCTCAGTACGTGAGATTGTACCCCACGAGCTGCCAC
ACGGCCTGCACTCTGCGCTTTGAGCTACTGGGCTGTGAGCTGAACGGATGCGCCAATCCCCTGGGCCTGAAGAATAACAG
CATCCCTGACAAGCAGATCACGGCCTCCAGCAGCTACAAGACCTGGGGCTTGCATCTCTTCAGCTGGAACCCCTCCTATG
CACGGCTGGACAAGCAGGGCAACTTCAACGCCTGGGTTGCGGGGAGCTACGGTAACGATCAGTGGCTGCAGGTGGACCTG
GGCTCCTCGAAGGAGGTGACAGGCATCATCACCCAGGGGGCCCGTAACTTTGGCTCTGTCCAGTTTGTGGCATCCTACAA
GGTTGCCTACAGTAATGACAGTGCGAACTGGACTGAGTACCAGGACCCCAGGACTGGCAGCAGTAAGATCTTCCCTGGCA
ACTGGGACAACCACTCCCACAAGAAGAACTTGTTTGAGACGCCCATCCTGGCTCGCTATGTGCGCATCCTGCCTGTAGCC
TGGCACAACCGCATCGCCCTGCGCCTGGAGCTGCTGGGCTGTGCTTCTAACTTTACTCAGTTCGTTCTCGTCGACAATGG
CGGAACTGGCGACGTGACTGTCGCCCCAAGCAACTTCGCTAACGGGGTCGCTGAATGGATCAGCTCTAACTCGCGTTCAC
AGGCTTACAAAGTAACCTGTAGCGTTCGTCAGAGCTCTGCGCAGAAACGCAAATACACCATCAAAGTCGAGGTGCCTAAA
GTGGCAACCCAGACTGTTGGTGGTGTAGAGCTTCCTGTAGCCGCATGGCGTTCGTACTTAAATATGGAACTAACCATTCC
AATTTTCGCTACGAATTCCGACTGCGAGCTTATTGTTAAGGCAATGCAAGGTCTCCTAAAAGATGGAAACCCGATTCCCT
CAGCAATCGCAGCAAACTCCGGCATCTACACCGGTATGGACTACAAGGATGACGATGACAAGGATTACAAAGACGACGAT
GATAAGGACTATAAGGATGATGACGACAAATGAGCTAGCGAAT

**Sequence S2. Sanger-confirmed sequence region of the PAC-pri-hsa-miR-146a-PAC construct.**

TAGCGTTTAAACGGGCCCTCTAGACTCGAGAAGGTACCTAATTGCCTAGAAAACATGAGGATCACCCATGTCTGCAGGTC
GACTCTAGAAACCGATGTGTATCCTCAGCTTTGAGAACTGAATTCCATGGGTTGTGTCAGTGTCAGACCTCTGAAATTCA
GTTCTTCAGCTGGGATATCTCTGTCATCGTCTGCAGGTCGACTCTAGAAAACATGAGGATCACCCATGTCTGCAGTATTC
CCGGGTTCATTAACCGGTCGGCCGCCTGTGCCTTCTAGTTGC
